# Potential effects of specific gut microbiota on periodontal disease: a two-sample bidirectional Mendelian randomization study

**DOI:** 10.3389/fmicb.2024.1322947

**Published:** 2024-01-19

**Authors:** Meng Xu, Qiang Shao, Yinglu Zhou, Yili Yu, Shuwei Wang, An Wang, Yida Cai

**Affiliations:** ^1^Department of Stomatology, Huashan Hospital, Fudan University, Shanghai, China; ^2^IT Department, Huashan Hospital, Fudan University, Shanghai, China; ^3^Nursing Department, Huashan Hospital, Fudan University, Shanghai, China; ^4^Dental Diseases Prevention and Treatment Center of Jiading District, Shanghai, China; ^5^Shanghai Jingan Dental Clinic, Shanghai, China

**Keywords:** gingivitis, periodontitis, periodontal disease, gut microbiota, gingival bleeding, Mendelian randomization

## Abstract

**Introduction:**

Periodontal disease (PD) presents a substantial global health challenge, encompassing conditions from reversible gingivitis to irreversible periodontitis, often culminating in tooth loss. The gut-oral axis has recently emerged as a focal point, with potential gut microbiota dysbiosis exacerbating PD.

**Methods:**

In this study, we employed a double-sample bidirectional Mendelian randomized (MR) approach to investigate the causal relationship between specific gut microbiota and periodontal disease (PD) and bleeding gum (BG) development, while exploring the interplay between periodontal health and the gut microenvironment. We performed genome-wide association studies (GWAS) with two cohorts, totalling 346,731 (PD and control) and 461,113 (BG and control) participants, along with data from 14,306 participants’ intestinal flora GWAS, encompassing 148 traits (31 families and 117 genera). Three MR methods were used to assess causality, with the in-verse-variance-weighted (IVW) measure as the primary outcome. Cochrane’s *Q* test, MR-Egger, and MR-PRESSO global tests were used to detect heterogeneity and pleiotropy. The leave-one-out method was used to test the stability of the MR results. An F-statistic greater than 10 was accepted for instrument exposure association.

**Results and conclusion:**

Specifically, *Eubacterium xylanophilum* and *Lachnoclostridium* were associated with reduced gum bleeding risk, whereas *Anaerotruncus*, *Eisenbergiella*, and *Phascolarctobacterium* were linked to reduced PD risk. Conversely, *Fusicatenibacter* was associated with an elevated risk of PD. No significant heterogeneity or pleiotropy was detected. In conclusion, our MR analysis pinpointed specific gut flora with causal connections to PD, offering potential avenues for oral health interventions.

## 1 Introduction

Periodontal disease (PD) is a global health issue encompassing two main categories: gingivitis and periodontitis. Gingival bleeding is a common clinical manifestation of both conditions (Chapple et al., 2017). Gingivitis, an initial stage of PD, is reversible (Tonetti et al., 2020). However, when gingivitis progresses into periodontitis, the disease leads to irreversible destruction of the supporting tissues surrounding the teeth, elevating the risk of bone loss and tooth loss (Chapple et al., 2017).

The prevalence of mild periodontitis affects approximately 20–50% of adults worldwide, whereas severe periodontitis afflicts around 10% and is a leading cause of adult tooth loss (Frencken et al., 2017; Papapanou et al., 2018; Genco and Sanz, 2020). Given its multifactorial nature, PD is linked not only to microbial infections but also to diabetes, cardiovascular diseases, metabolic disorders, obesity, rheumatoid arthritis, certain cancers, respiratory ailments, and cognitive impairments, including Alzheimer’s disease (Chapple et al., 2017; Genco and Sanz, 2020). To mitigate PD onset and progression, researchers have explored non-surgical and antibiotic alternatives (Chapple et al., 2017; Papapanou et al., 2018). Recently, novel avenues of research have involved inhibiting PD progression and its risk factors through interference with the gut–oral axis (Yang et al., 2015; Gatej et al., 2017; Wang et al., 2023).

The gut microbiota, which governs both the host’s intestinal metabolism and local/systemic immunity, plays a crucial role in health. Papageorgiou et al. (2017) established a connection between inflammatory bowel disease (IBD) and periodontitis, revealing that these conditions can interact to cause chronic inflammation and tissue damage in susceptible individuals. Both disorders involve abnormal immune responses and dysbiosis. Several researches on the role of the intestinal microbiota in periodontal disease have shown four critical elements. In terms of immune system interactions, the gut microbiota regulates host intestinal metabolites and modulates local/systemic immunity, hence impacting osteoclastogenesis and bone development (Ohlsson and Sjögren, 2015; Thaiss et al., 2016; Yan et al., 2016; Uchida et al., 2018; Amoroso et al., 2020; Jia et al., 2021; Sato et al., 2021; Shyu et al., 2021; Wang et al., 2023). According to research on microbial translocation, bacteria or their metabolites from the gut may translocate to the oral cavity via hematogenous pathways, contributing to the development or exacerbation of PD (Nagao et al., 2022; Basic and Dahlén, 2023; Sohn et al., 2023). Growing evidence suggests that disordered gut microbiota could contribute to varying degrees of periodontitis (Lourenςo et al., 2018; Zhang et al., 2020; Kawamoto et al., 2021). On the inflammation and systemic effects front, chronic inflammation, a common feature of both periodontal and intestinal diseases, may contribute to systemic health issues. Animal models have verified that gut dysbiosis impairs gut barrier function, disrupts oral microbiota, and exacerbates periodontitis-induced bone resorption via Th17/Treg imbalances (Yuan et al., 2023). Animal studies and omics analyses have associated gingivitis and periodontitis with gut microbiome shifts (Habashneh et al., 2012; Vavricka et al., 2013; Lourenςo et al., 2018; Li et al., 2020; Zhang et al., 2020; Kawamoto et al., 2021; Bao et al., 2022; Wu et al., 2022; Yuan et al., 2023). Concerning probiotics and prebiotics, researchers are exploring the potential use of probiotics and prebiotics to modulate the gut microbiome and, indirectly, impact periodontal health (Jia et al., 2019, 2021; Ausenda et al., 2023; Homayouni Rad et al., 2023). Nevertheless, these observations predominantly stem from observational cross-sectional and case–control studies and fall short of establishing causality.

Mendelian randomization (MR) leverages the natural randomization inherent in genetic single nucleotide polymorphisms (SNPs) as instrumental variables (IVs) to scrutinize observational data and uncover causal relationships in outcomes (Davey Smith and Ebrahim, 2003; Davey Smith et al., 2020). As high-throughput genome technology and genome-side association studies (GWAS) deepen our understanding of the disease, it becomes increasingly feasible to employ genetic data for extensive phenome research, a capability that can be harnessed for population-scale human microbiome data. Therefore, the primary goal of this study is to investigate the causal link between specific gut flora and PD. The aim is to unearth novel biomarkers, identify potentially effective clinical pathways for PD treatment, and provide evidence for future large-scale prospective cohort studies (Luo et al., 2023; Wang et al., 2023; Ye et al., 2023; Yu et al., 2023).

## 2 Materials and methods

### 2.1 Study design

**Figure 1:**
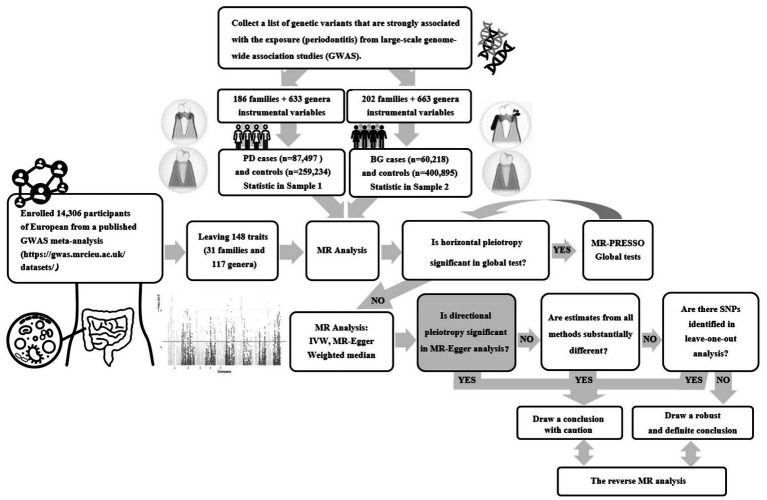
Flowchart of the two-sample bidirectional Mendelian randomization (MR) study. (PD, Periodontal Disease; BG, Bleeding Gums).

A two-sample bidirectional MR study was conducted using the UK Biobank and FinnGen Resource, this was a large-scale, population-based prospective cohort study. Figure 1 presents an overview of this research. Initially, we examined the causal link between gut microbial families/genera and the risk of PD or bleeding gums (BG). Then, we separately evaluated the reverse causation of gut microbial genera significantly associated with PD/BG. In this study, genetic variants served as instrumental variables (IVs) and adhered to three key assumptions: the reliability of genetic instruments (assumption 1), independence from unmeasured confounders (assumption 2), and the absence of horizontal pleiotropy (assumption 3).

### 2.2 Data sources and instruments

#### 2.2.1 Human gut microbiome

The present study retrieved summary-level human gut microbiota abundance data from a published GWAS meta-analysis^1^ (Supplementary Table S1). No additional ethics statement or consent was required for this study. The statistical data included 14,306 participants of European ancestry with two biological classifications, family and genus, that are closely associated with clinical diagnosis and treatment. Bacterial traits without specific species names (3 unknown families and 12 unknown genera) were excluded, leaving 148 traits (32 families and 118 genera). The *value of p* was set to <5 × 10^−6^ for IVs to obtain more explorable and sufficient results. According to the European reference panel (*r*^2^ < 0.001, distance = 10,000 kb), the linkage disequilibrium (LD) was investigated to clump SNPs and thus facilitate the independence of genetic variants. At least five SNPs were included in the meta-analysis to ensure the reliability of the MR. We aligned the retained variants with SNPs derived from outcome-association estimates for either periodontal disease (PD) or bleeding gums (BG). SNPs that did not present GWAS in the outcomes were systematically excluded or substituted with proxy SNPs demonstrating robust linkage disequilibrium (LD) (*r*^2^ > 0.8).

#### 2.2.2 Periodontal disease and bleeding gums

The diagnosis of “gingivitis and periodontal diseases” or “bleeding gums” for patients included in this study relied on rigorous histopathological assessments, encompassing self-reported information, official hospital diagnoses, and pertinent ICD10 codes. The resulting datasets were amalgamated for a comprehensive meta-analysis of both PD and BG. Summary-level results for BG and PD were extracted from the latest publicly accessible GWAS datasets, encompassing up to 461,113 and 346,731 consortium participants, respectively, after the exclusion of overlapping studies. The BG study’s genetic association data comprised 60,218 cases and 400,895 controls, with all cases and controls representing European populations aligned with outcome demographics. Similarly, the PD study’s genetic association data encompassed 87,497 cases and 259,234 controls, exclusively from European populations to match outcome demographics (Supplementary Table S1). Detailed methodologies, including data collection, participating cohorts, genotyping procedures, and data analysis, were comprehensively documented on their respective webpages.^2^^,^^3^ In the reverse MR analysis, the variants associated with PD were strictly executed with genome-wide significance (*p* < 5 × 10^−6^) and independence criteria (*r*^2^ < 0.001).

#### 2.2.3 Mendelian randomization and causal inference

The inverse-variance-weighted (IVW) method was employed as the primary MR analysis technique to assess the associations between specific gut microbial families/genera and the risk of PD/BG. The IVW method combines Wald estimators from individual SNPs to estimate the overall effects (Wang et al., 2022). The credibility of the IVW results relies on the assumption of the absence of horizontal pleiotropy for each SNP (Wang et al., 2022). To evaluate SNP heterogeneity, we conducted Cochran’s *Q* tests.

In cases where significant heterogeneity (*p* < 0.05) was observed, we applied a random effects IVW model, otherwise, a fixed effects IVW model was used (Wang et al., 2022). To ensure the robustness of our findings, we conducted sensitivity analyses, including analysis using the weighted median method, MR-Egger regression, and the MR pleiotropy residual sum and outlier (MR-PRESSO) test. Specifically, the weighted median estimator yields valid causal effect estimates when less than 50% of the information comes from invalid instruments (Bowden et al., 2016). In the MR-Egger regression, the *value of p* of the intercept term served as an indicator of directional pleiotropy (*p* < 0.05 was considered statistically significant) (Wang et al., 2022). The MR-PRESSO test was employed to detect and correct pleiotropic biases by identifying and removing outliers.


$$F=\frac{R^{2}\times \left(n-k-1\right)}{k\times \left(1-R^{2}\right)}\text{.}$$

To evaluate the potential impact of weak instrument bias on the effect estimates of causal associations, we assessed the strength of IVs using *F*-statistics. The *F*-statistics were computed using the following equation, with *R*^2^ representing the variance explained by the IVs (each gut microbiome) and n indicating the sample size (Feng et al., 2022):

The estimation of *R*^2^ employed minor allele frequency (MAF) and the β value, as determined by the following equation (Kurilshikov et al., 2021):


$$R^{2}=2\times MAF\times \left(1-MAF\right)\times \beta^{2}\text{.}$$

Furthermore, we investigated the assumption of the independence of IVs from confounding factors and outcomes for genome-wide significant associations (*p* < 5 × 10^−8^) by querying the PhenoScanner V2 website.^4^

If the results of all MR analyses reached nominal significance, we considered specific gut microbial genera as potentially associated with the risk of PD and BG. Subsequently, we conducted reverse direction MR analyses. All MR analyses were performed using R studio (version 2022.12.0 Build 353) and R (version 4.2.2) with the “Mendelian Randomization” and “MR-PRESSO” packages.

## 3 Results

### 3.1 The selection of instrumental variables

**Figure 2:**
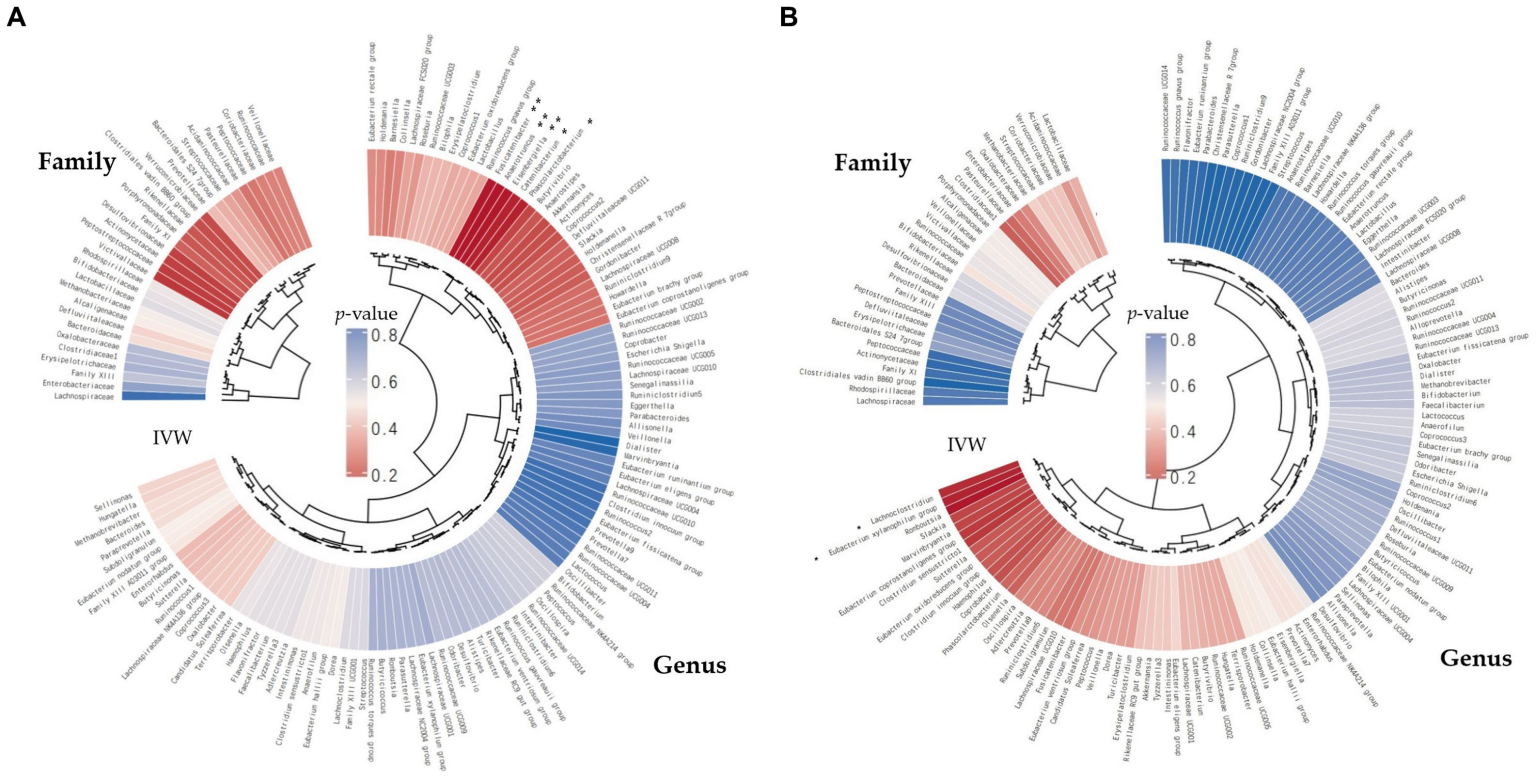
The inverse-variance-weighted (IVW) analyses pertaining to the relationships between gut microbial families/genera and the risk of periodontal disease (PD) **(A)** and the risk of bleeding gums (BG) **(B)**. The concentric circles denote the IVW analyses, with the innermost circle representing biological classifications. Gut microbiota classification is presented at the family and genus levels. The varying shades of color within the circles correspond to the magnitude of the *p*-values, as indicated by labels within the circle (**p < 0.01, *p < 0.05).

We screened 148 traits consisting of 31 families and 117 genera (Figure 2). Under the condition that the instrumental variable reaches the significance level of the whole locus (*p* < 5 × 10^−6^), after removing the known linkage disequilibrium effect of the flora, a total of 819 IVs for PD (186 families and 633 genera) and 865 IVs for BG (202 families and 663 genera) were retained. *F* was >10 for all instrumental variables, and the number of SNPs in each bacterial group was no less than 5.

### 3.2 Two-sample MR analysis

#### 3.2.1 Gingivitis and periodontal disease MR analysis

**Figure 3:**
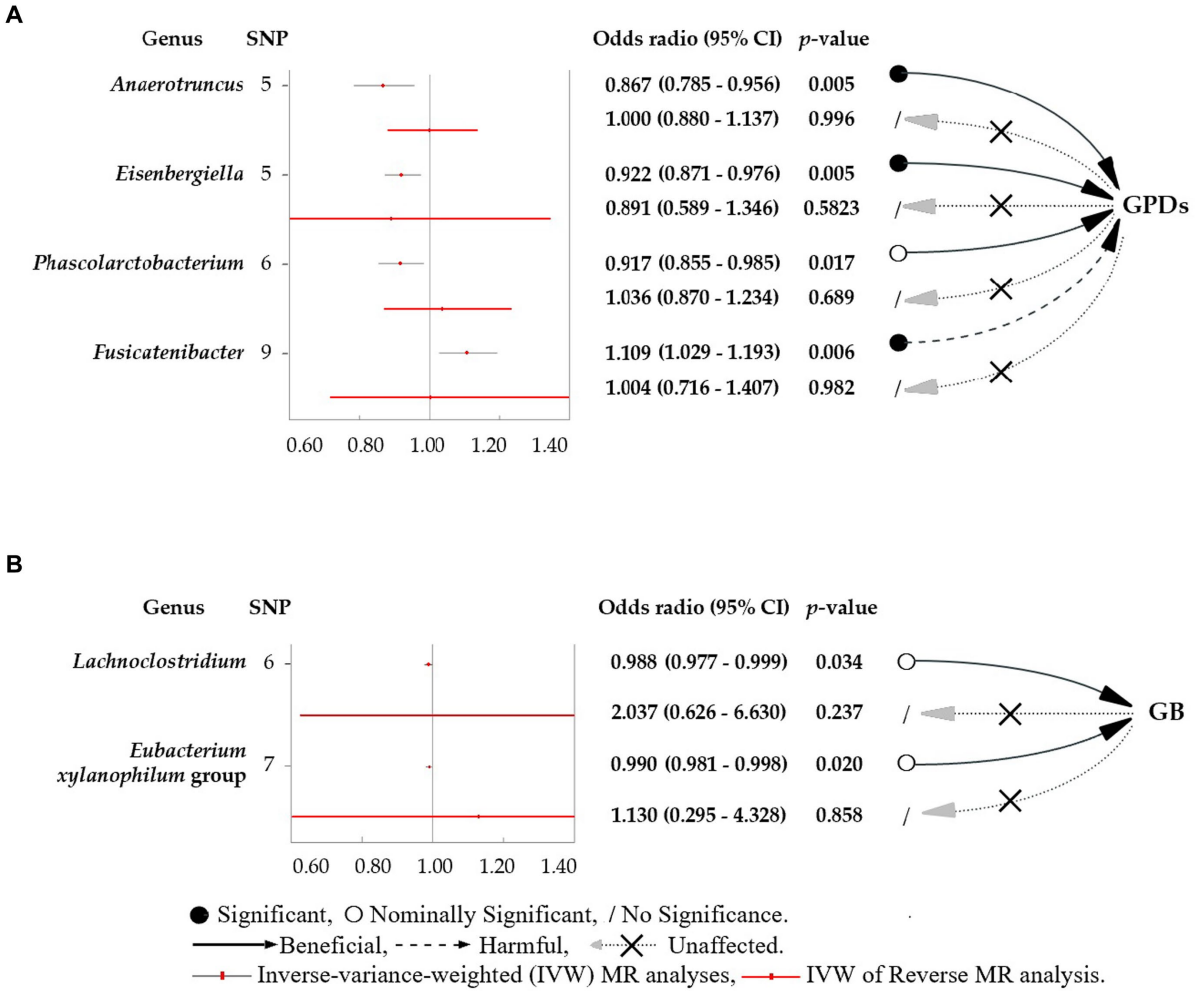
Forest plots featuring estimates derived from the inverse-variance-weighted (IVW) analysis. **(A)** The effects of SNPs for different gut microbial genera abundance levels on gingivitis and periodontal disease (PD). **(B)** The effects of SNPs for different gut microbial genera abundance levels on bleeding gums (BG). The red dots in the plots signify the IVW estimates, whereas the black and red bars represent the corresponding 95% confidence intervals (CIs) for the IVW estimates. An odds ratio (OR) > 1 signifies a risk factor, whereas an OR < 1 indicates a protective factor.

**Figure 4:**
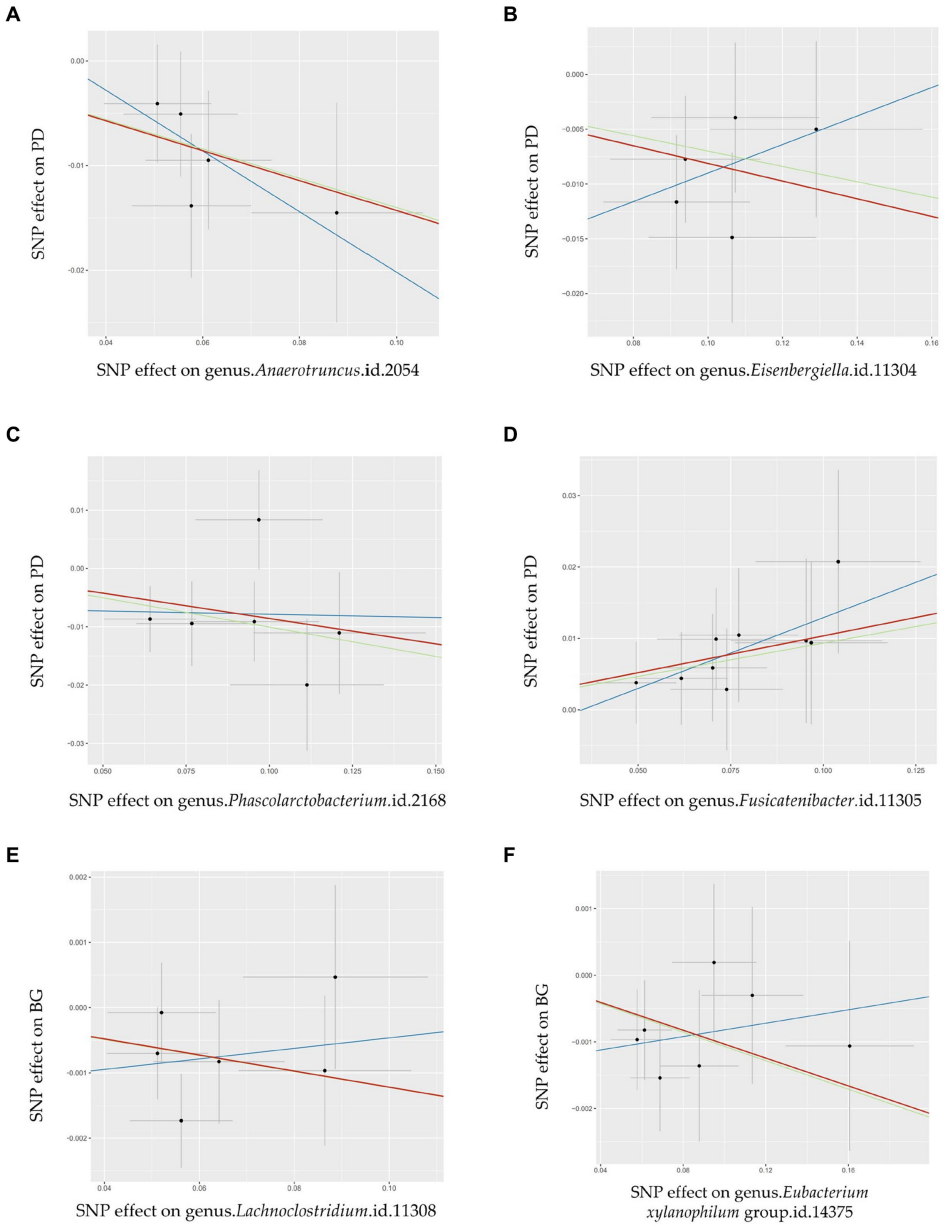
Scatter plots illustrating the Mendelian randomization (MR) estimates for the significant causality of 6 gut microbial genera and their impacts on the risk of periodontal disease (PD) or bleeding gums (BG). In subfigures **(A–D)**, we explore the causal effects of 4 gut microbial genera on PD, whereas subfigures **(E,F)** analyze the causal effect of 2 other gut microbial genera on BG. In these plots, upward-sloping diagonal lines signify positive correlations, indicating the facilitating effects of gut microbial genera on PD. Conversely, downward-sloping diagonal lines denote negative correlations, indicating the inhibitory impacts of gut microbiota on PD or BG. The horizontal and vertical lines within each plot represent the 95% confidence interval (CI) for each correlation. 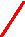 Inverse-variance-weighted (IVW), 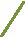 weighted median (WM), 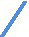 MR-Egger.

This study revealed four causal links between gut microbial genera and the risk of PD (Figure 3A). *Anaerotruncus* (*β* = −0.14, 95% CI: 0.79–0.96, SNP: 5) (Figure 4A), *Eisenbergiella* (*β* = −0.08, 95% CI: 0.87–0.98, SNP: 5) (Figure 4B), and *Phascolarctobacterium* (*β* = −0.09, 95% CI: 0.86–0.98, SNP: 6) (Figure 4C) demonstrated robust causal associations with reduced PD risk. In contrast, *Fusicatenibacter* (*β* = 0.10, 95% CI: 1.03–1.19, SNP: 9) (Figure 4D) was linked to an increased risk of PD. MR-Egger and MR-PRESSO tests detected no evidence of horizontal pleiotropy or outliers (*p* > 0.05). The results from Cochrane’s *Q*-test also indicated the absence of significant heterogeneity among the selected SNPs (*p* > 0.05). Our leave-one-out approach (Supplementary Figure S1A–D) confirmed that no single SNP dominated the positive results for the aforementioned microbiota.

#### 3.2.2 Bleeding gums

This study determined two causal links between gut microbial genera and the risk of BG (Figure 3B). The *Lachnoclostridium* (*β* = −0.01, 95% CI: 0.977–0.999, SNP: 6) (Figure 4E) and *Eubacterium xylanophilum* groups (*β* = −0.01, 95% CI: 0.981–0.998, SNP: 7) (Figure 4F) exhibited strong associations linked to a reduced risk of BG (Figure 3B). MR-Egger and MR-PRESSO tests (Supplementary Table S2) indicated the absence of horizontal pleiotropy or outliers (*p* > 0.05). Additionally, Cochrane’s *Q*-test results indicated no significant heterogeneity among the selected SNPs (*p* > 0.05). The leave-one-out method (Supplementary Figures S1E,F) confirmed that no single SNP could exert undue influence on the positive findings related to the aforementioned microbiota.

#### 3.2.3 Reverse MR analysis

Based on the above positive results, no discernible association between PD/BG and gut microbiota was observed when the six gut microbiota were considered as exposures (Figure 3; Supplementary Table S2). Similar outcomes for the association between PD/BG and the gut microbiota were consistently observed in the MR-PRESSO, MR-RAPS, and weighted median analyses (Supplementary Table S2). The weighted pattern approach yielded results that did not attain statistical significance.

## 4 Discussion

Our study represents a pioneering large-scale MR investigation, delving into the causal connection between gut microbiota and PD at the genetic level. Employing a two-sample MR approach, we not only identified gut microbiota associated with exacerbating or alleviating PD but also elucidated factors contributing to a decreased risk of gingival bleeding. Notably, *Anaerotruncus* and *Eisenbergiella* were linked to a reduced risk of PD, while *Fusicatenibacter* was associated with an increased risk of PD. This outcome was consistently corroborated by various analyses using IVW, WM, and MR-PRESSO, underscoring the robustness of our findings. These results were also validated in previous Mendelian studies with other databases (Luo et al., 2023; Ye et al., 2023). Discrepancies between this study and our findings may stem from variations in factors such as the stage of periodontitis, sex ratio, and ethnic composition of the sampled populations. In addition, the composition of gut microbiota can vary significantly among different populations (Luo et al., 2023; Ye et al., 2023).

The maintenance of intestinal stability by the microbiota involves the production of short-chain fatty acids (SCFAs), such as acetate, propionate, and butyrate. These SCFAs bolster host health and play important roles in cardiometabolic diseases (CMD) as PD risk factors, including obesity, type 2 diabetes (T2D), arteriosclerosis, and atherosclerosis (Den Besten et al., 2013). The microbiota also serves to suppress the proliferation of pathogenic microbes, thereby enhancing defense against pathogens. Moreover, the microbiota has an impact on immune system modulation, which guards against pro-inflammatory cytokines such as interleukin (IL)-6, IL-17, and tumour necrosis factor (TNF)-α and lessens the activation of T cells linked to the inflammatory response (Th17 and Th1) (Baldelli et al., 2021). Gut microbiota dysregulation, however, can lead to *Enterobacteriaceae* overgrowth, diminishing bacterial diversity, intestinal stability, and SCFA production, as well as increasing pro-inflammatory cytokines, weakening intestinal barrier function, and enhancing mucosal inflammation (Baldelli et al., 2021).

This study identified that *Anaerotruncus* and *Eisenbergiella* can significantly reduce the risk of PD. Some studies have suggested that *Anaerotruncus* and *Eisenbergiella* are linked to a reduced risk of PD. Anaerotruncus associated with increased tooth count and reduced periodontal pathogenic bacteria support its potential role in oral health (Li et al., 2020). *Eisenbergiella*’s negative correlation with prostaglandin E2 (PGE2), a marker upregulated in periodontitis patients, further supports its protective association (Lee et al., 2008; Li et al., 2020). Both genera produce butyrate, a key factor in maintaining intestinal homeostasis and possessing anti-inflammatory properties (Amir et al., 2014; Kelly et al., 2015; Togo et al., 2016; Silva et al., 2020). Butyrate serves as a regulator of transepithelial fluid transport, reducing mucosal inflammation and oxidative stress, enhancing the epithelial defense barrier, and controlling intestinal motility and visceral sensitivity (Canani et al., 2011). Although no direct studies have established the role of these bacteria in preventing PD, several studies have indicated their metabolic relevance. The abundance of *Anaerotruncus* in the gut decreases in individuals consuming high saturated fat/low fiber diets (Bailén et al., 2020). This genus includes species that ferment amino acids and certain carbohydrates, possibly contributing to the breakdown of mucins (Lau et al., 2006; Togo et al., 2019). *Eisenbergiella*’s abundance was increased in the feces of mice on a ketogenic diet (Ferrere et al., 2021). This increased abundance associated with neurodegenerative diseases (Noble et al., 2014; Stadlbauer et al., 2020) and depression (Fontana et al., 2020). Paradoxically, some studies found that *Eisenbergiella* could potentially exert a positive causal effect on periodontitis, thereby increasing the risk of this condition (Luo et al., 2023). Additionally, an increase in the abundance of the genus *Eisenbergiella* was linked to the exacerbation of certain chronic diseases, particularly those characterized by pro-inflammatory processes, which can include periodontitis (Bailén et al., 2020). As a genus, *Phascolarctobacterium* can nominally significantly reduce the risk of PD and produce acetate (as do *Anaerotruncus* and *Eisenbergiella*) (Mandaliya et al., 2021) and propionate (Mandaliya et al., 2021). Acetate can promotes microbiome-responsive IgA production (Wu et al., 2017; Takeuchi et al., 2021) and is effective in reducing inflammation by modulating immune responses (Mandaliya et al., 2021). Previous research linked *Phascolarctobacterium* to a reduced risk for PD through participation in host metabolism. For instance, a study from the Mayo Clinic Medical Centre emphasized highlighted its importance not only in helping people lose weight but also as a crucial regulator of intestinal group homeostasis when *Phascolarctobacterium* abundance was enhanced in adult intestinal flora. (Pedrogo et al., 2018).

*Eubacterium xylanophilum* and *Lachnoclostridium*, as butyrate producers (Uematsu and Hoshino, 1996; Oh et al., 2022), are nominally significantly associated with a potential reduction in the risk of BG. In mice fed a high-fat diet, dietary supplementation attenuated obesity and increased the gut abundance of butyrate-producing bacteria, including *Eubacterium xylanophilum* and *Lachnoclostridium* (Xiao et al., 2020; Zhang et al., 2020; Liu et al., 2021; Wei et al., 2021; Chen et al., 2022). Although the direct impact of these bacteria on BG has not been established, previous studies have suggested their relevance to host cardiometabolic disease and negative implications for obesity and T2D align with their potential protective role against PD (Nogal et al., 2021). Certain species of *Lachnoclostridium* efficiently converts choline into TMA (Cai et al., 2022), which is subsequently converted into trimethylamine-N-oxide (TMAO) in the liver (Jameson et al., 2016; Zhu et al., 2018). The TMAO pathway has been linked to cardiometabolic diseases such as obesity and T2D in humans (Dambrova et al., 2016; Schugar et al., 2017).

We consider that these five bacteria share a similar mechanism that may promote periodontal health. A healthy diet, particularly one decreasing saturated fat and promoting SCFAs production, might positively impact these bacteria, maintaining intestinal balance, reducing inflammation, and potentially influencing periodontal health. The result is a potential association with improved oral health and a reduced risk of PD and BG. This opens avenues for therapeutic targets in PD and gingival bleeding by modulating gut microbiota composition. (Li et al., 2021; Woelber et al., 2021; Woelber and Vach, 2022; Casarin et al., 2023). Further research is needed to clarify the causative role of these bacteria in preventing PD and explore therapeutic strategies that target these taxa.

We also observed a strong positive association between *Fusicatenibacter* and PD. *Fusicatenibacter*’s potential role in inducing periodontal disease may be linked to estrogen. *Fusicatenibacter* spp. exhibited significant increases in both the osteoporosis and Polycystic ovary syndrome (PCOS) groups, demonstrating a substantial positive correlation with postmenopausal time while showing a negative correlation with estrogen levels (Zhou et al., 2020; Yang et al., 2022). The intricate contributions of estrogen deficiency to alveolar bone loss remain elusive, however, multiple studies have endeavored to uncover these nuanced mechanisms. Eestrogen deficiency independently promote osteoclast survival and increased activity on its own, which accelerates bone resorption (Zhou et al., 2020). A noteworthy finding is the revealed inverse correlation between gut Fusicatenibacter abundance and postmenopausal osteopenia-enriched L-lysine (He et al., 2020). A MR study emphasized *Fusicatenibacter*’s recognition as a risk factor for bone mineral density (BMD) (Wang et al., 2023). This sheds light on the intricate mechanisms through which *Fusicatenibacter* may contribute to periodontal disease, implicating estrogen-related pathways and emphasizing the need for further investigation into these multifaceted associations. Additionally, *Fusicatenibacter*’s probable role in generating periodontal disease has been related to insomnia (Zhou et al., 2022). Genus *Fusicatenibacter* was dominant bacterial genus in patients with insomnia disorder, exhibiting increases in the abundance of the family *Prevotellaceae* and genus *Prevotella* (Zhou et al., 2022). Although *Fusicatenibacter* produces the beneficial SCFA (butyrate) and is inversely correlated with pro-inflammatory cytokines in human serum, the positive effects of this SCFA on the immune system may be counteracted by pro-inflammatory bacteria (such as those in the family *Prevotellaceae* and genus *Prevotella*) (Li et al., 2021). Furthermore, a high-collagen-peptide diet (collagen peptide from *tilapia nilotica* skin) caused a low abundance of *Fusicatenibacter* in rats (Mei et al., 2020).The link between periodontitis and the gut microbiota remains elusive, with several hypotheses proposed to elucidate this connection, including shared risk factors, immune activation and inflammation, metabolites and systemic effects, the brain–gut–oral axis feedback loop, shared nonspecific channels, and shared complex inflammatory pathways (Koppe et al., 2012; Vaziri et al., 2013; Ito and Yoshida, 2014; Chi et al., 2018; Chavakis et al., 2019; Jia et al., 2021; Malan-Muller et al., 2022; Newman and Kamada, 2022). In conclusion, although a mechanistic causal relationship between PD and the gut has not been established, the present MR findings remain valid. Oral administration may help degrade these metabolites and reduce specific gut flora, offering a potential therapeutic avenue for PD (Song et al., 2019; Wang et al., 2023).

Future research should focus on illuminating the mechanistic pathways through which specific gut microbiota influence oral health outcomes. Such research may involve investigating the role of gut-derived metabolites, immune responses, and inflammatory processes in oral health. Long-term prospective studies and clinical trials are also needed to validate the causal relationships identified in this MR analysis. Moreover, acknowledging the limitations of our study is imperative. Firstly, our study is exclusively based on data obtained from European populations, and therefore, it is crucial to exercise caution when extending these findings to other ethnic groups and races. Secondly, multiple MR analyses were conducted since the intestinal microbiome is categorized and summarized by various family/genus results. While stratified designs are commonly employed in clinical research, they are typically unadjusted for statistical significance levels (Luo et al., 2023; Ye et al., 2023). In this article, the threshold was set at a two-sided *p* < 0.05 (Luo et al., 2023; Ye et al., 2023). Thirdly, the inherent untestable assumptions in MR analysis emphasize the need for supplementary experimental and clinical validation studies to assess the clinical relevance of microbial species (Li et al., 2020). Fourthly, despite employing two independent authors for verification, the use of a PhenoScanner to mitigate the confounding effects of genetic variables may still introduce bias stemming from subjective factors, warranting cautious interpretation of the research outcomes. Moreover, the source data of our study did not provide information on the severity of the disease making it impossible to establish the relationship between intestinal flora and the severity of periodontitis cannot be given. Finally, the cross-sectional nature of the GWAS database precludes insights into the relationship between intestinal flora and the progression rate of periodontitis over time. A single investigation of the bacterial flora is insufficient to conclude this outcome. Given these limitations, we can only explore the causal associations between bacterial flora and periodontitis through the Mendelian randomization method. To contribute to a deeper understanding of the relationship between intestinal flora and the stage or progression rate of periodontitis, future studies should include the collection and documentation of clinical disease grade and progression rate while obtaining clinical samples.

## 5 Conclusion

Overall, our two-sample MR analysis identified specific gut microbiota taxa with causal links to the risk of PD and BG. *Anaerotruncus*, *Eisenbergiella*, and *Phascolarctobacterium* were identified as protective factors against PD, whereas *Fusicatenibacter* was implicated in promoting the occurrence of PD. These findings have implications for early disease diagnosis and the development of novel therapeutic strategies. Our study sets the stage for future largescale randomized controlled trials (RCTs) and may aid in predicting the outcomes of these trials. Future research should seek to determine the mechanistic pathways underpinning these causal relationships and explore the potential bidirectional interactions between oral health and the gut microbiota. Ultimately, a deeper understanding of these relationships has the potential to yield innovative approaches that enhance periodontal health and overall wellbeing.

## Data availability statement

Publicly available datasets were analyzed in this study. This data can be found at: https://gwas.mrcieu.ac.uk/datasets/; https://www.finngen.fi/fi.

## Author contributions

MX: Conceptualization, Methodology, Software, Validation, Writing – original draft. QS: Data curation, Formal analysis, Investigation, Methodology, Validation, Writing – original draft. YZ: Formal analysis, Investigation, Software, Validation, Writing – original draft. YY: Resources, Visualization, Writing – review & editing. SW: Conceptualization, Writing – review & editing. AW: Conceptualization, Funding acquisition, Supervision, Writing – review & editing. YC: Conceptualization, Project administration, Writing – review & editing.

## Footnotes

^1^https://gwas.mrcieu.ac.uk/datasets/ (Accessed January 20, 2023).

^2^https://www.finngen.fi/fi (Accessed January 20, 2023).

^3^https://gwas.mrcieu.ac.uk/ (Accessed January 20, 2023).

^4^http://www.phenoscanner.medschl.cam.ac.uk/ (Accessed January 20, 2023).
