# Supplementary material for: Potential effects of specific gut microbiota on periodontal disease: a two-sample bidirectional Mendelian randomization study
Source: Front Microbiol. 2024 Jan 19;15:1322947. doi: 10.3389/fmicb.2024.1322947 (PMC10834673; doi:10.3389/fmicb.2024.1322947)

Supplementary Material

# Supplementary Figures and Tables

## Supplementary Tables

**Supplementary Table S1.** Mendelian randomization analysis data source and details

| [**GWAS ID**](https://gwas.mrcieu.ac.uk/datasets/?gwas_id__icontains=&year__iexact=&trait__icontains=Periodontal+disease&consortium__icontains=&sort=-gwas_id) | [**Year**](https://gwas.mrcieu.ac.uk/datasets/?gwas_id__icontains=&year__iexact=&trait__icontains=Periodontal+disease&consortium__icontains=&sort=year) | [**Trait**](https://gwas.mrcieu.ac.uk/datasets/?gwas_id__icontains=&year__iexact=&trait__icontains=Periodontal+disease&consortium__icontains=&sort=-trait) | **Population** | [**Sample size**](https://gwas.mrcieu.ac.uk/datasets/?gwas_id__icontains=&year__iexact=&trait__icontains=Periodontal+disease&consortium__icontains=&sort=sample_size) | [**Number of SNPs**](https://gwas.mrcieu.ac.uk/datasets/?gwas_id__icontains=&year__iexact=&trait__icontains=Periodontal+disease&consortium__icontains=&sort=nsnp) |
| --- | --- | --- | --- | --- | --- |
| ukb-b-7872 Ben Elsworth | 2018 | Mouth/teeth dental problems: Bleeding gums | European | 461,113  (400,895 controls  and 60,218 cases) | 9,851,867 |
| K11_GINGIVITIS_  PERIODONTAL | 2023 | Gingivitis and periodontal diseases | European | 346,731  (259,234 controls  and 87,497 cases) | 20,169,620 |

**Supplementary Table S2.** Mendelian randomization causal effect estimates for six selected outcomes (PD: gingivitis and periodontal disease, BG: bleeding gums).

| PD **Exposure** | **Method** | **Positive** | | | **Negative** | | |
| --- | --- | --- | --- | --- | --- | --- | --- |
|  |  | **OR** | **OR 95% CI** | ***p*-value** | **OR** | **OR 95% CI** | ***p*-value** |
| **Gut microbiota abundance**  **(Genus *Anaerotruncus* id.2054)**  **ebi-a-GCST90016967**  **nSNP = 5** | IVW | 0.867 | 0.785 – 0.957 | 0.005 | 1.000 | 0.880 – 1.137 | 0.996 |
|  | MR-Egger | 0.748 | 0.405 – 1.383 | 0.423 | 1.430 | 0.668 – 3.06 | 0.384 |
|  | WM | 0.869 | 0.766 – 0.987 | 0.030 | 0.993 | 0.863 – 1.14 | 0.917 |
|  | MR-PRESSO | 0.867 | 0.788 – 0.952 | 0.007 | 1.022 | 0.907 – 1.15 | 0.730 |
| **Gut microbiota abundance**  **(Genus *Eisenbergiella* id.11304)**  **ebi-a-GCST90016991**  **nSNP = 5** | IVW | 0.922 | 0.871 – 0.976 | 0.005 | 0.891 | 0.589 – 1.346 | 0.5823 |
|  | MR-Egger | 1.139 | 0.709 – 1.828 | 0.628 | 1.473 | 0.276 – 7.87 | 0.663 |
|  | WM | 0.932 | 0.867 – 1.002 | 0.059 | 0.854 | 0.502 – 1.45 | 0.559 |
|  | MR-PRESSO | 0.950 | 0.899 – 1.004 | 0.145 | 0.914 | 0.683 – 1.22 | 0.559 |
| **Gut microbiota abundance**  **(Genus *Phascolarctobacterium* id.2168)**  **ebi-a-GCST90017043**  **nSNP = 6** | IVW | 0.918 | 0.855 – 0.985 | 0.017 | 1.036 | 0.870 – 1.234 | 0.689 |
|  | MR-Egger | 0.989 | 0.688 – 1.420 | 0.955 | 0.347 | 0.140 – 0.86 | 0.052 |
|  | WM | 0.904 | 0.823 – 0.993 | 0.036 | 0.995 | 0.846 – 1.17 | 0.955 |
|  | MR-PRESSO | 0.923 | 0.853 – 0.998 | 0.074 | 1.050 | 0.894 – 1.23 | 0.560 |
| **Gut microbiota abundance**  **(Genus *Fusicatenibacter* id.11305)**  **ebi-a-GCST90017011**  **nSNP = 9** | IVW | 1.109 | 1.030 – 1.193 | 0.006 | 1.004 | 0.716 – 1.407 | 0.982 |
|  | MR-Egger | 1.219 | 0.866 – 1.717 | 0.293 | 0.283 | 0.098 – 0.82 | 0.048 |
|  | WM | 1.098 | 1.002 – 1.203 | 0.045 | 1.256 | 0.835 – 1.89 | 0.274 |
|  | MR-PRESSO | 1.092 | 1.044 – 1.143 | 0.002 | 0.926 | 0.705 – 1.22 | 0.590 |
|  |  |  |  |  |  |  |  |
| **BG Exposure** | **Method** | **Positive** | | | **Negative** | | |
|  |  | **OR** | **OR 95% CI** | ***p*-value** | **OR** | **OR 95% CI** | ***p*-value** |
| **Gut microbiota abundance**  **(Genus *Lachnoclostridium* id.11308)**  **ebi-a-GCST90017020**  **nSNP = 6** | IVW | 0.988 | 0.977 – 0.999 | 0.034 | 2.037 | 0.626 – 6.630 | 0.237 |
|  | MR-Egger | 1.008 | 0.954 – 1.065 | 0.789 | 98.879 | 0.105 – 93439 | 0.199 |
|  | WM | 0.988 | 0.973 – 1.003 | 0.105 | 1.315 | 0.243 – 7.13 | 0.751 |
|  | MR-PRESSO | 0.988 | 0.977 – 0.999 | 0.061 | 1.576 | 0.534 – 4.65 | 0.416 |
| **Gut microbiota abundance**  **(genus *Eubacterium xylanophilum* group id.14375)**  **ebi-a-GCST90017006**  **nSNP = 7** | IVW | 0.990 | 0.981 – 0.998 | 0.0198 | 1.130 | 0.295 – 4.328 | 0.858 |
|  | MR-Egger | 1.005 | 0.978 – 1.033 | 0.734 | 0.110 | 0.001 – 21.6 | 0.420 |
|  | WM | 0.989 | 0.978 – 1.000 | 0.071 | 0.817 | 0.156 – 4.28 | 0.811 |
|  | MR-PRESSO | 0.993 | 0.983 – 1.002 | 0.109 | 1.187 | 0.357 – 3.95 | 0.781 |

## Supplementary Figures

**Supplementary Figure S1.** MR leave-one-out sensitivity analysis for the effects of SNPs on PD and BG for different gut microbiota abundance levels. The X-axis shows the MR leave-one-out sensitivity analysis of different gut microbiota abundance levels for PD and BG. The Y-axis shows the impact analysis of the SNP leave-one-out method on PD or BG. (PD: gingivitis and periodontal disease, BG: bleeding gums, MR: Mendelian randomization).

1. **Genus.*Anaerotruncus*.id.2054 MR leave-one-out sensitivity analysis for “|| id: ebi-a-GCST90016967’ on ‘GINGIVITIS & PERIODONTAL”
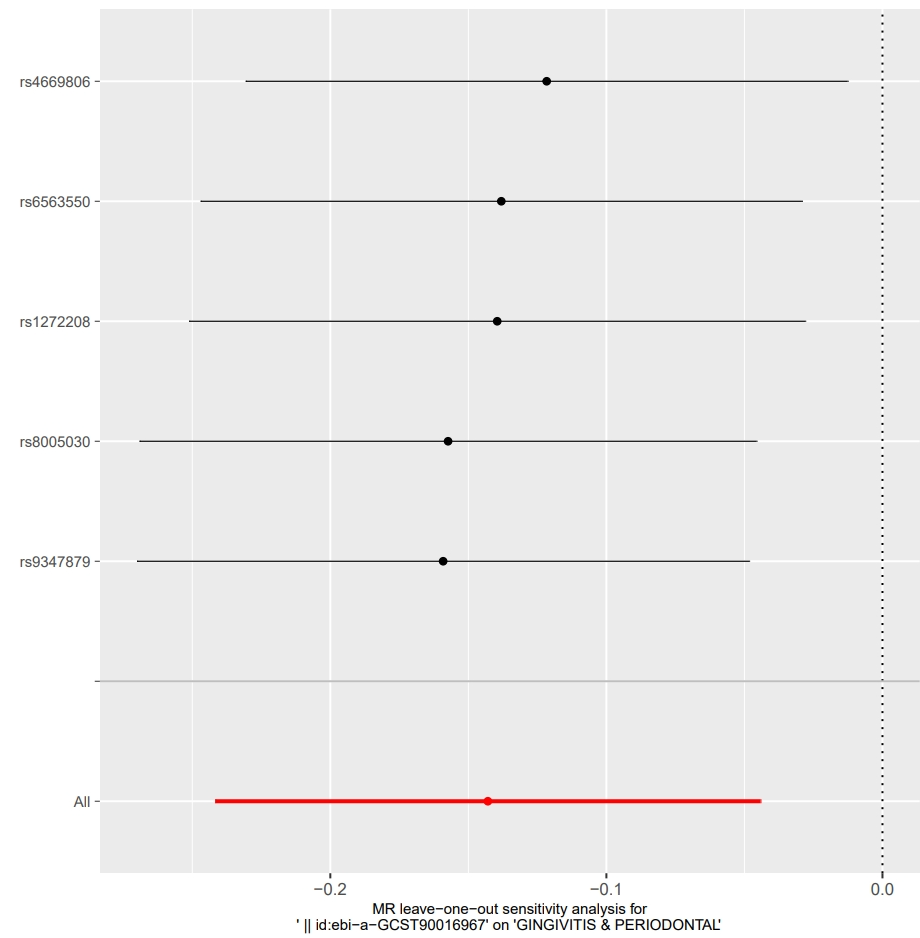
**
2. **Genus.Eisenbergiella.id.11304 MR leave-one-out sensitivity analysis for “|| id: ebi-a-GCST90016991’ on ‘GINGIVITIS & PERIODONTA”**
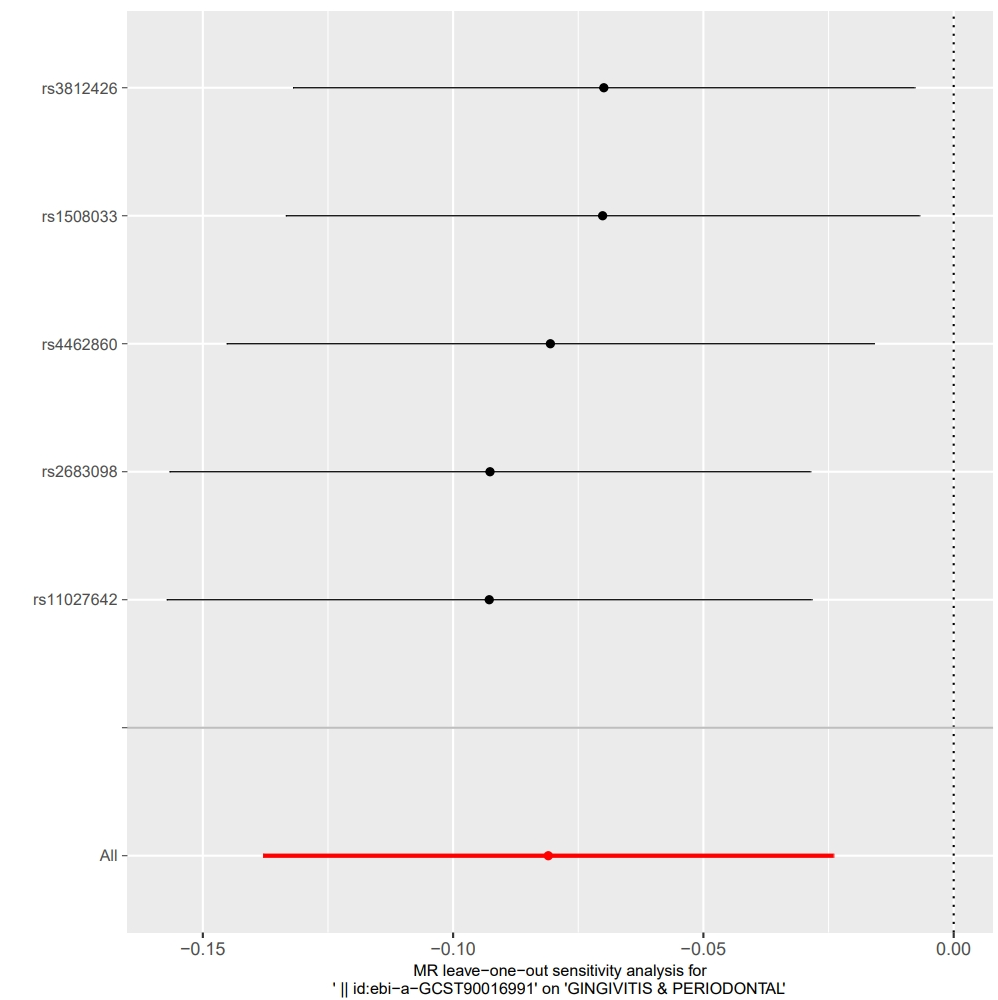

3. **Genus.*Phascolarctobacterium.*id.2168 MR leave-one-out sensitivity analysis for “|| id: ebi-a-GCST90017043’ on ‘GINGIVITIS & PERIODONTAL”**
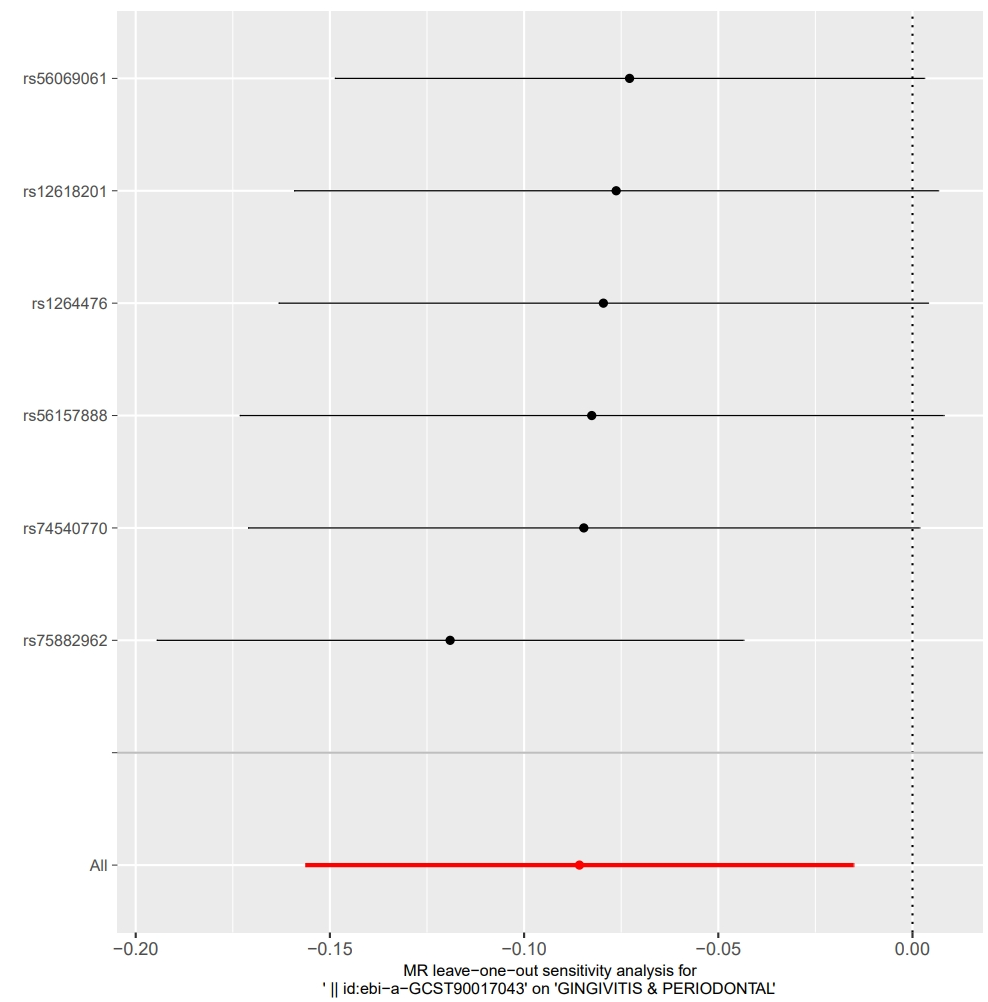

4. **Genus.*Fusicatenibacter.*id.11305 MR leave-one-out sensitivity analysis for ‘‘|| id: ebi-a-GCST90017011’ on ‘GINGIVITIS & PERIODONTAL’**
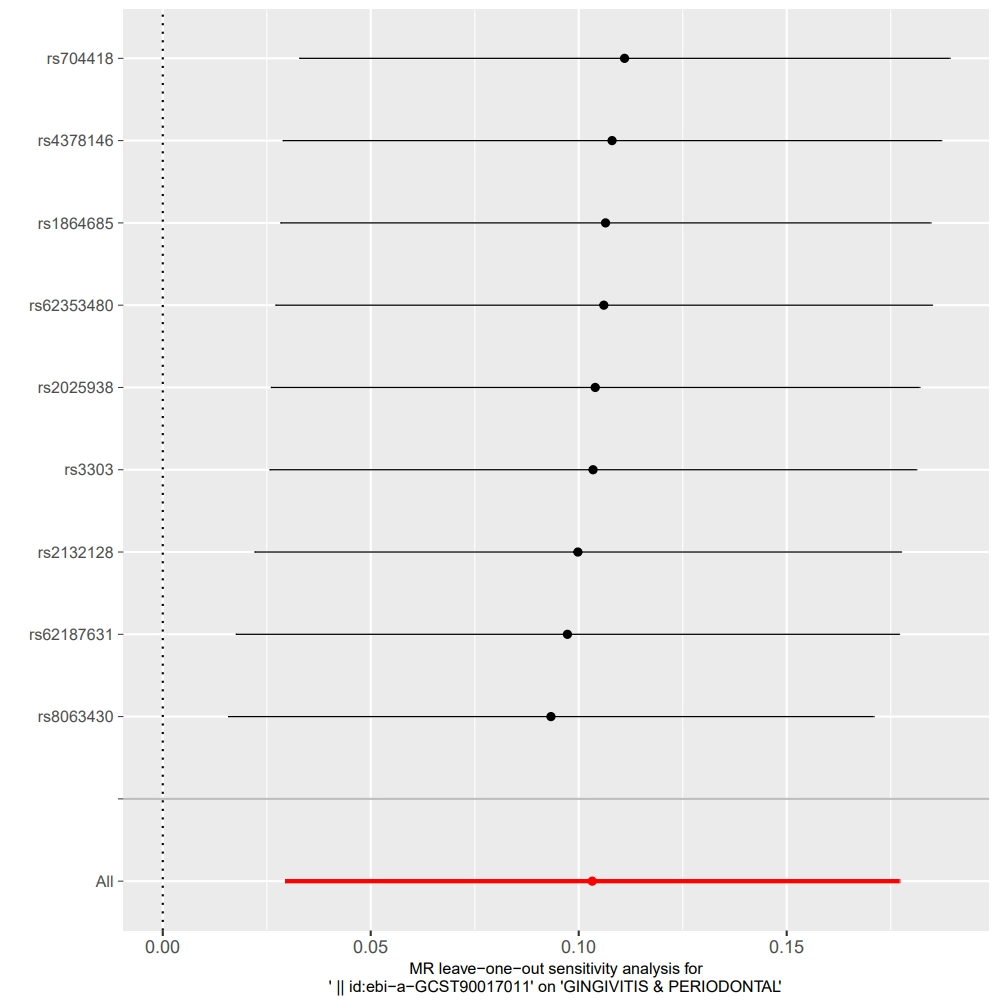

5. **Genus.*Lachnoclostridium*.id.11308 MR leave-one-out sensitivity analysis for ‘‘|| id: ebi-a-GCST90017020’ on ‘Mouth/teeth dental problems: Bleeding gums || id: ukb-b-7872’**
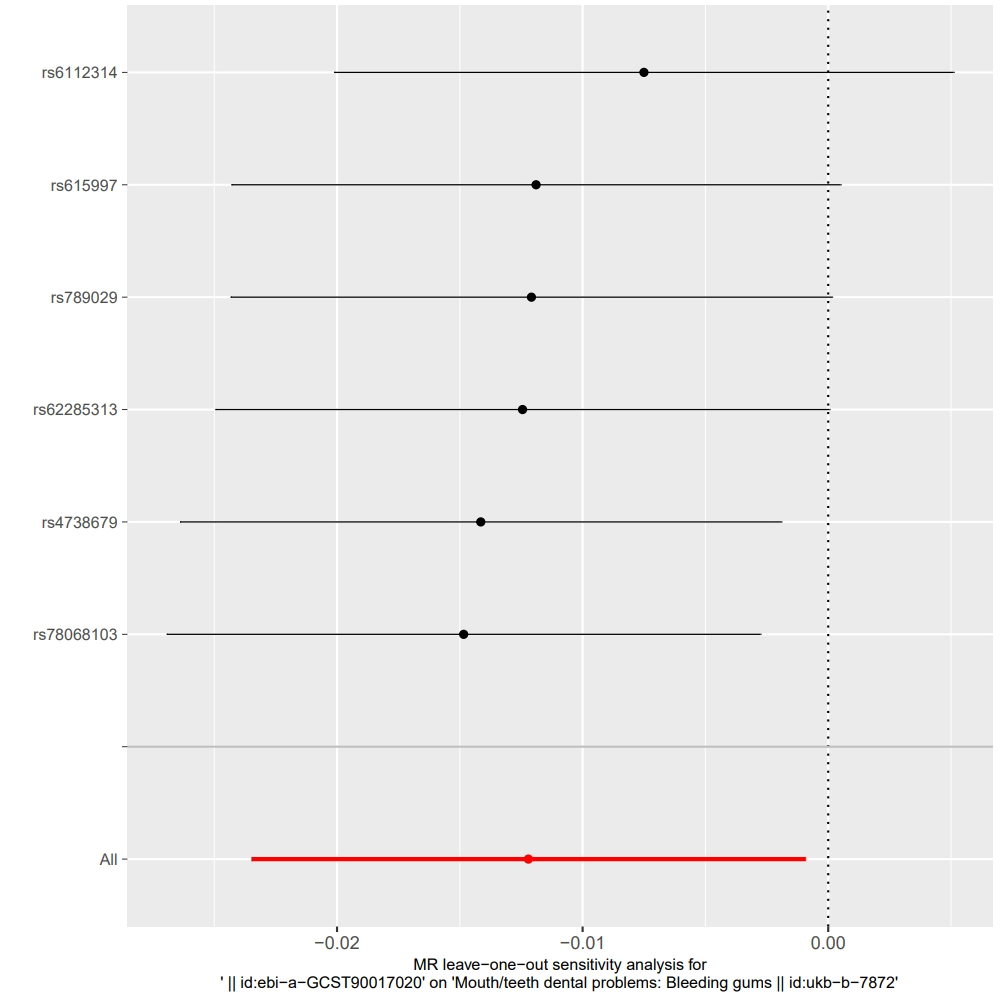

6. **Genus.*Eubacterium xylanophilum group.*id.14375 MR leave-one-out sensitivity analysis for ‘|| id: ebi-a-GCST90017006’ on ‘Mouth/teeth dental problems: Bleeding gums || id: ukb-b-7872’**


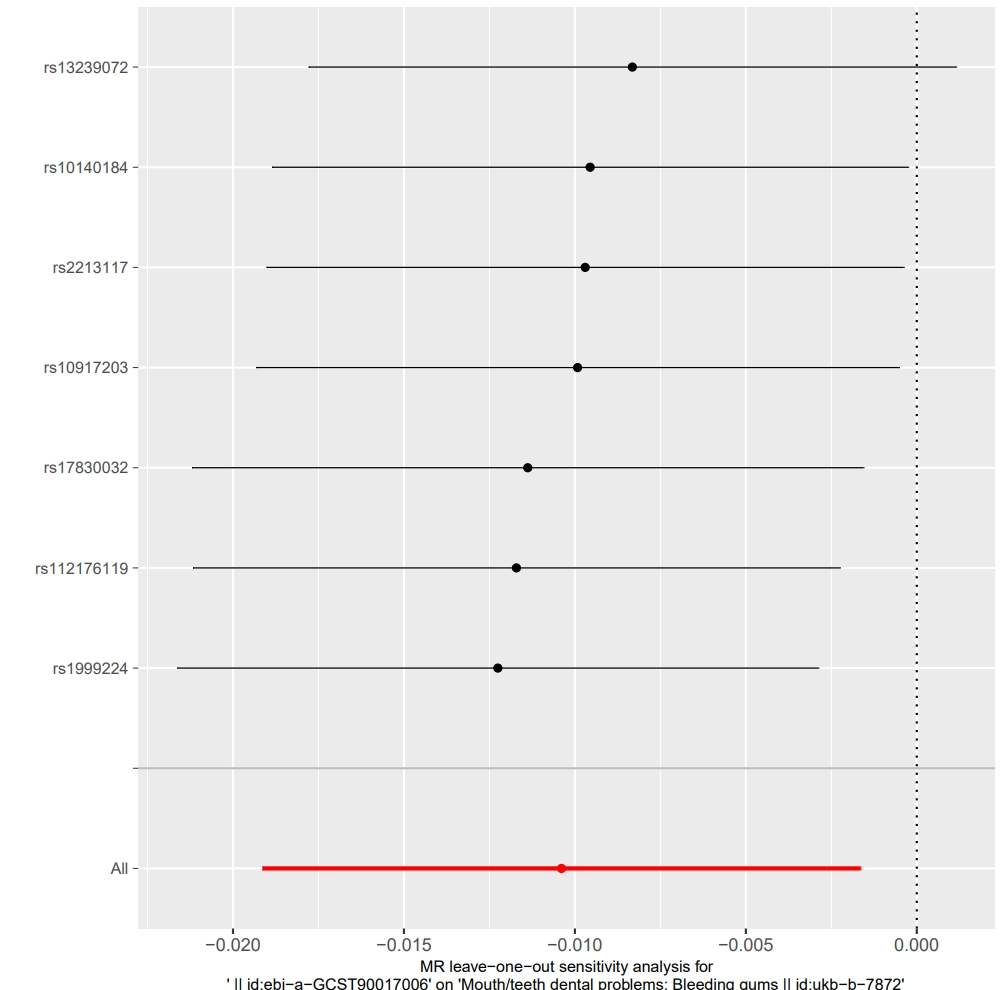

Supplement: Supplementary file 1 [file Data_Sheet_1.docx]
