# Supplementary material for: Potential effects of specific gut microbiota on periodontal disease: a two-sample bidirectional Mendelian randomization study
Source: Front Microbiol. 2024 Jan 19;15:1322947. doi: 10.3389/fmicb.2024.1322947 (PMC10834673; doi:10.3389/fmicb.2024.1322947)
Supplement: Supplementary file 2 [file Data_Sheet_2.docx]

library("devtools")

library(MRInstruments) # Introduction: https://github.com/mrcieu/mrinstruments

library(MRPracticals) # Introduction: MR Radial

library(TwoSampleMR)

library(MRPRESSO)

library(mr.raps)

library(writexl)

library(tidyverse)

library(RadialMR)

library(ieugwasr) # outcomes sum

# README

# local: C:\Users\85773\Documents\MR

## case:

# ** - finngen_R9_K11_GINGIVITIS_PERIODONTAL

# - finngen_R9_K11_PERIODON_CHRON.gz

# - finngen_R9_K11_PERIODON_CHRON_COMPL.gz

## Two-SAMPLE MR, Multi-variable MR

## databases:

# *************************************************************************

# Exposure: Phenotype, families (31) + genus (117)

# *************************************************************************

ao <- available_outcomes()

ao <- readxl::read_xlsx("GWAS_ALL_outcomes.xlsx")

write_xlsx(ao,"GWAS_ALL_outcomes.xlsx")

# filter: ==, >, >= etc; &, |, !, xor(); is.na(); between(), near()

ao_dental <- ao %>%

filter(id == 'ebi-a-GCST90016924' #Gut microbiota abundance (family Acidaminococcaceae id.2166)

|id == 'ebi-a-GCST90016925' #Gut microbiota abundance (family Actinomycetaceae id.421)

|id == 'ebi-a-GCST90016926' #Gut microbiota abundance (family Alcaligenaceae id.2875)

|id == 'ebi-a-GCST90016927' #Gut microbiota abundance (family Bacteroidaceae id.917)

|id == 'ebi-a-GCST90016928' #Gut microbiota abundance (family Bacteroidales S24 7group id.11173)

|id == 'ebi-a-GCST90016929' #Gut microbiota abundance (family Bifidobacteriaceae id.433)

|id == 'ebi-a-GCST90016930' #Gut microbiota abundance (family Christensenellaceae id.1866)

|id == 'ebi-a-GCST90016931' #Gut microbiota abundance (family Clostridiaceae1 id.1869)

|id == 'ebi-a-GCST90016932' #Gut microbiota abundance (family Clostridiales vadin BB60 group id.11286)

|id == 'ebi-a-GCST90016933' #Gut microbiota abundance (family Coriobacteriaceae id.811)

|id == 'ebi-a-GCST90016934' #Gut microbiota abundance (family Defluviitaleaceae id.1924)

|id == 'ebi-a-GCST90016935' #Gut microbiota abundance (family Desulfovibrionaceae id.3169)

|id == 'ebi-a-GCST90016936' #Gut microbiota abundance (family Enterobacteriaceae id.3469)

|id == 'ebi-a-GCST90016937' #Gut microbiota abundance (family Erysipelotrichaceae id.2149)

|id == 'ebi-a-GCST90016938' #Gut microbiota abundance (family Family XI id.1936)

|id == 'ebi-a-GCST90016939' #Gut microbiota abundance (family Family XIII id.1957)

|id == 'ebi-a-GCST90016940' #Gut microbiota abundance (family Lachnospiraceae id.1987)

|id == 'ebi-a-GCST90016941' #Gut microbiota abundance (family Lactobacillaceae id.1836)

|id == 'ebi-a-GCST90016942' #Gut microbiota abundance (family Methanobacteriaceae id.121)

|id == 'ebi-a-GCST90016943' #Gut microbiota abundance (family Oxalobacteraceae id.2966)

|id == 'ebi-a-GCST90016944' #Gut microbiota abundance (family Pasteurellaceae id.3689)

|id == 'ebi-a-GCST90016945' #Gut microbiota abundance (family Peptococcaceae id.2024)

|id == 'ebi-a-GCST90016946' #Gut microbiota abundance (family Peptostreptococcaceae id.2042)

|id == 'ebi-a-GCST90016947' #Gut microbiota abundance (family Porphyromonadaceae id.943)

|id == 'ebi-a-GCST90016948' #Gut microbiota abundance (family Prevotellaceae id.960)

|id == 'ebi-a-GCST90016949' #Gut microbiota abundance (family Rhodospirillaceae id.2717)

|id == 'ebi-a-GCST90016950' #Gut microbiota abundance (family Rikenellaceae id.967)

|id == 'ebi-a-GCST90016951' #Gut microbiota abundance (family Ruminococcaceae id.2050)

|id == 'ebi-a-GCST90016952' #Gut microbiota abundance (family Streptococcaceae id.1850)

|id == 'ebi-a-GCST90016956' #Gut microbiota abundance (family Veillonellaceae id.2172)

|id == 'ebi-a-GCST90016957' #Gut microbiota abundance (family Verrucomicrobiaceae id.4036)

|id == 'ebi-a-GCST90016958') #Gut microbiota abundance (family Victivallaceae id.2255)

# *********************************************************************

# offline - outcome extraction

# Comments

# - SNP = rsIDS

# - effect_allele = alt

# - other_allele = ref

# - beta = beta

# - se = sebeta

# - pval = pval

# *********************************************************************

# ************************* Gut Microbiota (family) ****************************

# extract_Gut microbiota_GWAS_instruments

dental_exp_dat_1 <- extract_instruments(outcomes = c('ebi-a-GCST90016924',

'ebi-a-GCST90016925',

'ebi-a-GCST90016926',

'ebi-a-GCST90016927',

'ebi-a-GCST90016928',

'ebi-a-GCST90016929',

'ebi-a-GCST90016930',

'ebi-a-GCST90016931',

'ebi-a-GCST90016932',

'ebi-a-GCST90016933',

'ebi-a-GCST90016934',

'ebi-a-GCST90016935',

'ebi-a-GCST90016936',

'ebi-a-GCST90016937',

'ebi-a-GCST90016938',

'ebi-a-GCST90016939',

'ebi-a-GCST90016940',

'ebi-a-GCST90016941',

'ebi-a-GCST90016942',

'ebi-a-GCST90016943',

'ebi-a-GCST90016944',

'ebi-a-GCST90016945',

'ebi-a-GCST90016946',

'ebi-a-GCST90016947',

'ebi-a-GCST90016948',

'ebi-a-GCST90016949',

'ebi-a-GCST90016950',

'ebi-a-GCST90016951',

'ebi-a-GCST90016952',

'ebi-a-GCST90016956',

'ebi-a-GCST90016957',

'ebi-a-GCST90016958'),

p1 = 5e-06,

clump = TRUE,

r2 = 0.001,

kb = 10000,

access_token = ieugwasr::check_access_token(),

force_server = FALSE )

#Counting the SNPs Number for each Microbiota

dental_exp_SNP_NUM <- summary(as.factor(dental_exp_dat_1$id.exposure)) #show the clumped SNPs NUMB >5, pval 5e-6

write.csv(dental_exp_SNP_NUM, 'dental_exp_SNP_NUM[family].csv')

head(dental_exp_dat_1,10) # 236 obs of 15 var; not all exposure SNP>= 5

write.csv(dental_exp_dat_1, 'dental_exp_dat_1[family].csv') # Gut microbiota_GWAS_instruments for dental problems

#1. input: finn_local, 'GINGIVITIS_PERIODONTAL + PERIODON_CHRON'

dental_GWAS_1 <- read.delim("finngen_R9_K11_GINGIVITIS_PERIODONTAL.gz") # txt | txt.gz | tsv.gz

head(dental_GWAS_1) # finngen_R9_K11_GINGIVITIS_PERIODONTAL

#dental_GWAS_2 <- read.delim("finngen_R9_K11_PERIODON_CHRON.gz") # txt | txt.gz | tsv.gz

#head(dental_GWAS_2) # finngen_R9_K11_PERIODON_CHRON

#dental_GWAS_3 <- read.delim("finngen_R9_K11_PERIODON_CHRON_COMPL.gz") # txt | txt.gz | tsv.gz

#head(dental_GWAS_3) # finngen_R9_K11_PERIODON_CHRON_COMPL

#2. Merge data

##2.1 Exposures(1): dental_exp_dat_1 ['dat_1' standing for the family of microbiotas]

##2.2 Exposures(2): dental_exp_dat_2 ['dat_2' standing for the genus of microbiotas]

##2.3 Outcomes(1): dental_GWAS_1 [GWAS_1 standing for GINGIVITIS_PERIODONTAL: case ~ 87497 vs. ctl ~ 259,234]

##2.4 Outcomes(2): dental_GWAS_2 [GWAS_2 standing for dental problems: Bleeding gums: case ~ 60,218 vs. ctl ~ 400,895]

##2.3 Outcomes(1)

head(dental_exp_dat_1) # exposure clumped

head(dental_GWAS_1) # outcome GINGIVITIS_PERIODONTAL

meg_gut_dental.family <- merge(dental_exp_dat_1, dental_GWAS_1, by.x = "SNP", by.y = "rsids")

head(meg_gut_dental.family)

# Table head modify and select

##2.3 Outcomes(1)

meg_gut_dental_modi.family <- meg_gut_dental.family %>%

mutate(effect_allele = alt, other_allele = ref, se = sebeta, eaf = af_alt) %>%

dplyr::select(SNP,effect_allele,other_allele,beta,pval,se,eaf)

head(meg_gut_dental_modi.family) # obs: 221 on condition IV pval<5e-6

write.csv(meg_gut_dental_modi.family,"outcomes_gut_to_dental[family].csv")

# merge GWAS exposure (gut microbiota.family) and outcome (dental problems)

# [Attention!] remove first col in "outcomes_gut_to_dental[family].csv"

#3. Outcomes DATA

##2.3 Outcomes(1)

gut_dental_out_dat.family <- read_outcome_data(snps = dental_exp_dat_1$SNP,

filename = "outcomes_gut_to_dental[family].csv",

sep = ",", # .csv format

snp_col = "SNP",

beta_col = "beta",

se_col = "se",

effect_allele_col = "effect_allele",

other_allele_col = "other_allele",

pval_col = "pval",

eaf_col = "eaf")

head(gut_dental_out_dat.family)

# outcome modified

gut_dental_out_dat.family$outcome <- 'GINGIVITIS & PERIODONTAL'

view(gut_dental_out_dat.family) # obs: 220 comments Duplicated SNPs present

write.csv(gut_dental_out_dat.family,"gut_dental_out_dat[family].csv")

#=============================================================

##2.3 Outcomes(2) -- ukb-b-7872 bleeding gum -- online

#=============================================================

gut_dental_out_dat.family2 <- extract_outcome_data(snps = dental_exp_dat_1$SNP, # dental_exp_dat_1$SNP -- microbiota

outcomes = 'ukb-b-7872', # 'ukb-b-7872' == Mouth/teeth dental problems: Bleeding gums

maf_threshold = 0.01, # proxies = F,

access_token = NULL)

head(gut_dental_out_dat.family2)

write.csv(gut_dental_out_dat.family2,'gut_dental_out_family2.csv')

# Other key points:

# - http://www.phenoscanner.medschl.cam.ac.uk/

# - Sample overlapping

# - Proxy search: https://snipa.helmholtz-muenchen.de/snipa3/

# - Numb. of outcome SNP ?

# - Harmonization: 1. Exposure-SNP vs. Outcome-SNP; 2. EAF palindromic SNP; 3. Exclude incompatible SNP

dental_dat_anal.family <- harmonise_data(dental_exp_dat_1, gut_dental_out_dat.family)

write.csv(dental_dat_anal.family,'harmonise_gut_dental_01[family].csv')

# add outcome_se

dental_dat_anal.family$se.outcome=sqrt(((dental_dat_anal.family$beta.outcome)^2)/qchisq(dental_dat_anal.family$pval.outcome,1,lower.tail=F))

# revised id

write_xlsx(dental_dat_anal.family,"gut_to_dental_finn_se_01[family].xlsx")

dental_dat_anal.family %>% dplyr::select(pval.outcome) %>% summary() ## SNP to outcome check

# all results

res_all_dental_anal.family <- mr(dental_dat_anal.family,method_list = c("mr_ivw","mr_egger_regression","mr_weighted_median"))

#simple ivw results

res_ivw_dental_anal.family <- mr(dental_dat_anal.family,method_list = c("mr_ivw"))

# odds ratios

res_ivw_or_dental_anal.family <- generate_odds_ratios(res_ivw_dental_anal.family)

res_all_or_dental_anal.family <- generate_odds_ratios(res_all_dental_anal.family)

## ******************************************

## ------------- statistics -----------------

## -------------- [family] ------------------

## ------------- outcomes(1) ----------------

## dental_dat_anal.family <- harmonise_data()

## ******************************************

# heterogeneity statistics

hetero_dental_dat_anal.family <- mr_heterogeneity(dental_dat_anal.family)

mr_heterogeneity(dental_dat_anal.family, method_list=c("mr_egger_regression", "mr_ivw"))

write.csv(hetero_dental_dat_anal.family,'hetero_dental_dat_anal.family.csv')

# pleiotropy

pleio_dental_dat_anal.family <- mr_pleiotropy_test(dental_dat_anal.family)

pleio_dental_dat_anal.family

write.csv(pleio_dental_dat_anal.family,'pleio_dental_dat_anal.family.csv')

#leave-one-out

single_dental_dat_anal.family <- mr_singlesnp(dental_dat_anal.family)

loo_dental_dat_anal.family <- mr_leaveoneout(dental_dat_anal.family)

write.csv(single_dental_dat_anal.family,"single_dental_dat_anal.family.csv")

write.csv(loo_dental_dat_anal.family,"loo_dental_dat_anal.family.csv")

# heterogeneity statistics

# mr_presso((BetaOutcome = "Y_effect", BetaExposure = "E1_effect",

#SdOutcome = "Y_se", SdExposure = "E1_se", OUTLIERtest = TRUE,

#DISTORTIONtest = TRUE, data = SummaryStats, NbDistribution = 1000, SignifThreshold = 0.05)

# data cleaning

head(res_all_or_dental_anal.family)

# Scatter plot

# mr_ivw, mr_egger_regression, mr_weighted_median

res_subgroup_dental_dat_anal.family <- mr(dental_dat_anal.family,method_list = c("mr_ivw","mr_egger_regression","mr_weighted_median"))

p1_dental_dat_anal.family <- mr_scatter_plot(res_subgroup_dental_dat_anal.family, dental_dat_anal.family)

# plot frame [1]

# p1[[1]]

# 5 IN 1

length(p1_dental_dat_anal.family)

#p1_dental_dat_anal.family

# export pdf plot

for (i in 1:length(p1_dental_dat_anal.family)) {

# create PDF

pdf(paste0("plot_", i, ".pdf"))

# Print plots

print(p1_dental_dat_anal.family[[i]])

# turn off the sequence

dev.off()

}

# Forest plot

res_single_dental_dat_anal.family <- mr_singlesnp(dental_dat_anal.family)

head(res_single_dental_dat_anal.family)

p2_forest_dental_dat_anal.family <- mr_forest_plot(res_single_dental_dat_anal.family)

length(p2_forest_dental_dat_anal.family)

# p2_forest_dental_dat_anal.family

# export pdf plot

for (i in 1:length(p2_forest_dental_dat_anal.family)) {

# create PDF

pdf(paste0("Forest_plot_", i, ".pdf"))

# Print plots

print(p2_forest_dental_dat_anal.family[[i]])

# turn off the sequence

dev.off()

}

# Leave-one-out plot - sensitivity

res_loo_dental_dat_anal.family <- mr_leaveoneout(dental_dat_anal.family)

p3_loo_dental_dat_anal.family <- mr_leaveoneout_plot(res_loo_dental_dat_anal.family)

# p3_loo_dental_dat_anal.family

# export pdf plot

for (i in 1:length(p3_loo_dental_dat_anal.family)) {

# create PDF

pdf(paste0("loo_plot_", i, ".pdf"))

# Print plots

print(p3_loo_dental_dat_anal.family[[i]])

# turn off the sequence

dev.off()

}

# funnel plot

# res_single_dental_dat_anal.family <- mr_singlesnp(dental_dat_anal.family)

res_single_dental_dat_anal.family <- mr_singlesnp(dental_dat_anal.family)

p4_funnel_dental_dat_anal.family <- mr_funnel_plot(res_single_dental_dat_anal.family)

# p4_funnel_dental_dat_anal.family

# export pdf plot

for (i in 1:length(p4_funnel_dental_dat_anal.family)) {

# create PDF

pdf(paste0("funnel_", i, ".pdf"))

# Print plots

print(p4_funnel_dental_dat_anal.family[[i]])

# turn off the sequence

dev.off()

}

## ******************************************

## ******************************************

# Exclude the exposure ID labels from the exposure column

# res_ivw_dental_anal.family <- split_outcome(res_ivw_dental_anal.family)

res_ivw_or_dental_anal.family <- split_outcome(res_ivw_or_dental_anal.family)

# this sorts results by decreasing effect size (largest effect at top of the plot)

# Forest plot

# res_plot1_dental_anal.family <- res_ivw_dental_anal.family

res_plot1_dental_anal.family <- res_ivw_or_dental_anal.family

head(res_plot1_dental_anal.family)

plot1_dental_anal.family <- forest_plot_1_to_many(

res_plot1_dental_anal.family,

b = "or",

se = "se",

exponentiate = TRUE,

trans = "log2",

ao_slc = FALSE,

lo = 0.8, # beta upci

up = 2, # beta loci

col1_width = 2,

TraitM = "exposure", # exposure/outcome

col_text_size = 3,

shape_points = 15,

xlab = ""

)

plot1_dental_anal.family

# odds ratios

# res_ivw_or_dental_anal.family <- generate_odds_ratios(res_ivw_dental_anal.family)

# res_all_or_dental_anal.family <- generate_odds_ratios(res_all_dental_anal.family)

# write.csv(res_ivw_dental_anal.family,'res_ivw_dental_anal[family].csv')

write.csv(res_ivw_or_dental_anal.family,'res_ivw_or_dental_anal[family].csv')

write.csv(res_all_or_dental_anal.family,'res_all_or_dental_anal[family].csv')

# ==================================

##2.4 Outcomes(2)

# ==================================

dental_dat_anal.family2 <- harmonise_data(dental_exp_dat_1, gut_dental_out_dat.family2)

# harmonise_data('instruments', 'extract_outcome')

write.csv(dental_dat_anal.family2,'harmonise_gut_dental_01[family2].csv')

# add outcome_se

dental_dat_anal.family2$se.outcome=sqrt(((dental_dat_anal.family2$beta.outcome)^2)/qchisq(dental_dat_anal.family2$pval.outcome,1,lower.tail=F))

# revised id

write_xlsx(dental_dat_anal.family2,"gut_to_dental_finn_se_01[family2].xlsx")

dental_dat_anal.family2 %>% dplyr::select(pval.outcome) %>% summary() ## SNP to outcome check

# all results

res_all_dental_anal.family2 <- mr(dental_dat_anal.family2,method_list = c("mr_ivw","mr_egger_regression","mr_weighted_median"))

#simple ivw results

res_ivw_dental_anal.family2 <- mr(dental_dat_anal.family2,method_list = c("mr_ivw"))

# odds ratios

res_ivw_or_dental_anal.family2 <- generate_odds_ratios(res_ivw_dental_anal.family2)

res_all_or_dental_anal.family2 <- generate_odds_ratios(res_all_dental_anal.family2)

# write.csv(res_ivw_dental_anal.family,'res_ivw_dental_anal[family].csv')

write.csv(res_ivw_or_dental_anal.family2,'res_ivw_or_dental_anal[family2].csv')

write.csv(res_all_or_dental_anal.family2,'res_all_or_dental_anal[family2].csv')

## ******************************************

## ------------- statistics -----------------

## -------------- [family] ------------------

## ------------- outcomes(2) ----------------

## dental_dat_anal.family <- harmonise_data()

## ******************************************

# heterogeneity statistics

hetero_dental_dat_anal.family2 <- mr_heterogeneity(dental_dat_anal.family2)

mr_heterogeneity(dental_dat_anal.family2, method_list=c("mr_egger_regression", "mr_ivw"))

write.csv(hetero_dental_dat_anal.family2,'hetero_dental_dat_anal.family2.csv')

# pleiotropy

pleio_dental_dat_anal.family2 <- mr_pleiotropy_test(dental_dat_anal.family2)

pleio_dental_dat_anal.family2

write.csv(pleio_dental_dat_anal.family2,'pleio_dental_dat_anal.family2.csv')

#leave-one-out

single_dental_dat_anal.family2 <- mr_singlesnp(dental_dat_anal.family2)

loo_dental_dat_anal.family2 <- mr_leaveoneout(dental_dat_anal.family2)

write.csv(single_dental_dat_anal.family2,'single_dental_dat_anal.family2.csv')

write.csv(loo_dental_dat_anal.family2,'loo_dental_dat_anal.family2.csv')

# heterogeneity statistics

# mr_presso((BetaOutcome = "Y_effect", BetaExposure = "E1_effect",

#SdOutcome = "Y_se", SdExposure = "E1_se", OUTLIERtest = TRUE,

#DISTORTIONtest = TRUE, data = SummaryStats, NbDistribution = 1000, SignifThreshold = 0.05)

# data cleaning

head(res_all_or_dental_anal.family2)

# Scatter plot

# mr_ivw, mr_egger_regression, mr_weighted_median

res_subgroup_dental_dat_anal.family2 <- mr(dental_dat_anal.family2,method_list = c("mr_ivw","mr_egger_regression","mr_weighted_median"))

p1_dental_dat_anal.family2 <- mr_scatter_plot(res_subgroup_dental_dat_anal.family2, dental_dat_anal.family2)

# plot frame [1]

# p1[[1]]

# 5 IN 1

length(p1_dental_dat_anal.family2)

#p1_dental_dat_anal.family

# export pdf plot

for (i in 1:length(p1_dental_dat_anal.family2)) {

# create PDF

pdf(paste0("plot_family2_", i, ".pdf"))

# Print plots

print(p1_dental_dat_anal.family2[[i]])

# turn off the sequence

dev.off()

}

# Forest plot

res_single_dental_dat_anal.family2 <- mr_singlesnp(dental_dat_anal.family2)

head(res_single_dental_dat_anal.family2)

p2_forest_dental_dat_anal.family2 <- mr_forest_plot(res_single_dental_dat_anal.family2)

length(p2_forest_dental_dat_anal.family2)

# p2_forest_dental_dat_anal.family

# export pdf plot

for (i in 1:length(p2_forest_dental_dat_anal.family2)) {

# create PDF

pdf(paste0("Forest_plot_", i, ".pdf"))

# Print plots

print(p2_forest_dental_dat_anal.family2[[i]])

# turn off the sequence

dev.off()

}

# Leave-one-out plot - sensitivity

res_loo_dental_dat_anal.family2 <- mr_leaveoneout(dental_dat_anal.family2)

p3_loo_dental_dat_anal.family2 <- mr_leaveoneout_plot(res_loo_dental_dat_anal.family2)

# p3_loo_dental_dat_anal.family2

# export pdf plot

for (i in 1:length(p3_loo_dental_dat_anal.family2)) {

# create PDF

pdf(paste0("loo_plot_", i, ".pdf"))

# Print plots

print(p3_loo_dental_dat_anal.family2[[i]])

# turn off the sequence

dev.off()

}

# funnel plot

# res_single_dental_dat_anal.family <- mr_singlesnp(dental_dat_anal.family)

res_single_dental_dat_anal.family2 <- mr_singlesnp(dental_dat_anal.family2)

p4_funnel_dental_dat_anal.family2 <- mr_funnel_plot(res_single_dental_dat_anal.family2)

# p4_funnel_dental_dat_anal.family

# export pdf plot

for (i in 1:length(p4_funnel_dental_dat_anal.family2)) {

# create PDF

pdf(paste0("funnel_", i, ".pdf"))

# Print plots

print(p4_funnel_dental_dat_anal.family2[[i]])

# turn off the sequence

dev.off()

}

## ******************************************

## ******************************************

# Exclude the exposure ID labels from the exposure column

res_ivw_dental_anal.family2 <- split_outcome(res_ivw_or_dental_anal.family2)

# this sorts results by decreasing effect size (largest effect at top of the plot)

# Forest plot

res_plot1_dental_anal.family2 <- res_ivw_dental_anal.family2

plot1_dental_anal.family2 <- forest_plot_1_to_many(

res_plot1_dental_anal.family2,

b = "or",

se = "se",

exponentiate = TRUE,

trans = "log2",

ao_slc = FALSE,

lo = 0.9, # beta upci

up = 1.1, # beta loci

col1_width = 2,

TraitM = "exposure", # exposure/outcome

col_text_size = 3,

shape_points = 15,

xlab = ""

)

plot1_dental_anal.family2

#write.csv(res_ivw_dental_anal.family2,'res_ivw_dental_anal[family2].csv')

write.csv(res_ivw_or_dental_anal.family2,'res_ivw_or_dental_anal[family2].csv')

write.csv(res_all_or_dental_anal.family2,'res_all_or_dental_anal[family2].csv')

# -----------------------------------------------------------------------------

# ************************* Gut Microbiota (genus) ****************************

# -----------------------------------------------------------------------------

# extract_Gut microbiota_GWAS_instruments

# Gut Microbiota (genus)

dental_exp_dat_2_1 <- extract_instruments(outcomes = c('ebi-a-GCST90016959',

'ebi-a-GCST90016960',

'ebi-a-GCST90016961',

'ebi-a-GCST90016962',

'ebi-a-GCST90016963',

'ebi-a-GCST90016964',

'ebi-a-GCST90016965',

'ebi-a-GCST90016966',

'ebi-a-GCST90016967',

'ebi-a-GCST90016968',

'ebi-a-GCST90016969',

'ebi-a-GCST90016970',

'ebi-a-GCST90016971',

'ebi-a-GCST90016972',

'ebi-a-GCST90016973',

'ebi-a-GCST90016974',

'ebi-a-GCST90016975',

'ebi-a-GCST90016976',

'ebi-a-GCST90016977',

'ebi-a-GCST90016978',

'ebi-a-GCST90016979',

'ebi-a-GCST90016980',

'ebi-a-GCST90016981',

'ebi-a-GCST90016982',

'ebi-a-GCST90016983',

'ebi-a-GCST90016984',

'ebi-a-GCST90016985',

'ebi-a-GCST90016986',

'ebi-a-GCST90016987',

'ebi-a-GCST90016988',

'ebi-a-GCST90016989',

'ebi-a-GCST90016990',

'ebi-a-GCST90016991',

'ebi-a-GCST90016992',

'ebi-a-GCST90016993',

'ebi-a-GCST90016994',

'ebi-a-GCST90016995',

'ebi-a-GCST90016996',

'ebi-a-GCST90016997',

'ebi-a-GCST90016998',

'ebi-a-GCST90016999',

'ebi-a-GCST90017000',

'ebi-a-GCST90017001',

'ebi-a-GCST90017002'),

p1 = 5e-06,

clump = TRUE,

r2 = 0.001,

kb = 10000,

access_token = ieugwasr::check_access_token(),

force_server = FALSE )

head(dental_exp_dat_2_1)

write.csv(dental_exp_dat_2_1,'dental_exp_dat_2_1.csv')

dental_exp_dat_2_2 <- extract_instruments(outcomes = c('ebi-a-GCST90017003',

'ebi-a-GCST90017004',

'ebi-a-GCST90017005',

'ebi-a-GCST90017006',

'ebi-a-GCST90017007',

'ebi-a-GCST90017008',

'ebi-a-GCST90017009',

'ebi-a-GCST90017010',

'ebi-a-GCST90017011',

'ebi-a-GCST90017012',

'ebi-a-GCST90017013',

'ebi-a-GCST90017014',

'ebi-a-GCST90017015',

'ebi-a-GCST90017016',

'ebi-a-GCST90017017',

'ebi-a-GCST90017018',

'ebi-a-GCST90017019',

'ebi-a-GCST90017020',

'ebi-a-GCST90017029',

'ebi-a-GCST90017021',

'ebi-a-GCST90017022',

'ebi-a-GCST90017023',

'ebi-a-GCST90017024',

'ebi-a-GCST90017025',

'ebi-a-GCST90017026',

'ebi-a-GCST90017027',

'ebi-a-GCST90017028',

'ebi-a-GCST90017030',

'ebi-a-GCST90017031'),

p1 = 5e-06,

clump = TRUE,

r2 = 0.001,

kb = 10000,

access_token = ieugwasr::check_access_token(),

force_server = FALSE )

head(dental_exp_dat_2_2)

write.csv(dental_exp_dat_2_2,'dental_exp_dat_2_2.csv')

dental_exp_dat_2_3 <- extract_instruments(outcomes = c('ebi-a-GCST90017032',

'ebi-a-GCST90017033',

'ebi-a-GCST90017034',

'ebi-a-GCST90017035',

'ebi-a-GCST90017036',

'ebi-a-GCST90017037',

'ebi-a-GCST90017038',

'ebi-a-GCST90017039',

'ebi-a-GCST90017040',

'ebi-a-GCST90017041',

'ebi-a-GCST90017042',

'ebi-a-GCST90017043',

'ebi-a-GCST90017044',

'ebi-a-GCST90017045',

'ebi-a-GCST90017046',

'ebi-a-GCST90017047',

'ebi-a-GCST90017048',

'ebi-a-GCST90017049',

'ebi-a-GCST90017050',

'ebi-a-GCST90017051',

'ebi-a-GCST90017052',

'ebi-a-GCST90017053',

'ebi-a-GCST90017054',

'ebi-a-GCST90017055',

'ebi-a-GCST90017056',

'ebi-a-GCST90017057',

'ebi-a-GCST90017058',

'ebi-a-GCST90017059',

'ebi-a-GCST90017060',

'ebi-a-GCST90017061',

'ebi-a-GCST90017064',

'ebi-a-GCST90017065',

'ebi-a-GCST90017066',

'ebi-a-GCST90017062',

'ebi-a-GCST90017063',

'ebi-a-GCST90017067',

'ebi-a-GCST90017068',

'ebi-a-GCST90017069',

'ebi-a-GCST90017070',

'ebi-a-GCST90017071',

'ebi-a-GCST90017072',

'ebi-a-GCST90017073',

'ebi-a-GCST90017074',

'ebi-a-GCST90017075',

'ebi-a-GCST90017088'),

p1 = 5e-06,

clump = TRUE,

r2 = 0.001,

kb = 10000,

access_token = ieugwasr::check_access_token(),

force_server = FALSE )

head(dental_exp_dat_2_3)

write.csv(dental_exp_dat_2_3,'dental_exp_dat_2_3.csv')

# merge data

dental_exp_dat_2_test <- full_join(dental_exp_dat_2_1,dental_exp_dat_2_2)

dental_exp_dat_2 <- full_join(dental_exp_dat_2_test, dental_exp_dat_2_3)

# Gut [genus] microbiota_GWAS_instruments for dental problems

write.csv(dental_exp_dat_2, 'dental_exp_dat_2[genus].csv')

dental_exp_SNP_NUM_2_1 <- summary(as.factor(dental_exp_dat_2_1$id.exposure))

dental_exp_SNP_NUM_2_2 <- summary(as.factor(dental_exp_dat_2_2$id.exposure))

dental_exp_SNP_NUM_2_3 <- summary(as.factor(dental_exp_dat_2_3$id.exposure))

# merge data

write.csv(dental_exp_SNP_NUM_2_1, 'dental_exp_SNP_NUM_2_1[genus].csv')

write.csv(dental_exp_SNP_NUM_2_2, 'dental_exp_SNP_NUM_2_2[genus].csv')

write.csv(dental_exp_SNP_NUM_2_3, 'dental_exp_SNP_NUM_2_3[genus].csv')

head(dental_exp_dat_2,10) # 789 obs of 15 var; not all exposure SNP>= 5

# 'dental_exp_dat_2[genus].csv'

# ==================================

##2.3 Outcomes(1) GINGIVITIS_PERIODONTAL

##2.4 Outcomes(2) Bleeding Gums

# ==================================

#1. input: finn_local

dental_GWAS_1 <- read.delim("finngen_R9_K11_GINGIVITIS_PERIODONTAL.gz") # txt | txt.gz | tsv.gz

head(dental_GWAS_1) # finngen_R9_K11_GINGIVITIS_PERIODONTAL

#dental_GWAS_2 <- read.delim("finngen_R9_K11_PERIODON_CHRON.gz") # txt | txt.gz | tsv.gz

#head(dental_GWAS_2) # finngen_R9_K11_PERIODON_CHRON

#dental_GWAS_3 <- read.delim("finngen_R9_K11_PERIODON_CHRON_COMPL.gz") # txt | txt.gz | tsv.gz

#head(dental_GWAS_3) # finngen_R9_K11_PERIODON_CHRON_COMPL

#2. Merge data [gut Mirob genus]

# ==================================

##2.3 Outcomes(1) GINGIVITIS_PERIODONTAL

# ==================================

meg_gut_genus_dental.genus <- merge(dental_exp_dat_2, dental_GWAS_1, by.x = "SNP", by.y = "rsids")

head(meg_gut_genus_dental.genus) # 760 obs

# Table head modify and select

meg_gut_genus_dental_modi.genus <- meg_gut_genus_dental.genus %>%

mutate(effect_allele = alt, other_allele = ref, se = sebeta, eaf = af_alt) %>%

dplyr::select(SNP,effect_allele,other_allele,beta,pval,se,eaf)

head(meg_gut_genus_dental_modi.genus) # obs: 760 on condition IV pval<5e-8

write.csv(meg_gut_genus_dental_modi.genus,"outcomes_gut_genus_to_dental[genus].csv")

#3. Outcomes DATA

gut_dental_out_dat.genus <- read_outcome_data(snps = dental_exp_dat_2$SNP, # genus -- exposure 'dental_exp_dat_2'

filename = "outcomes_gut_genus_to_dental[genus].csv",

sep = ",", # .csv format

snp_col = "SNP",

beta_col = "beta",

se_col = "se",

effect_allele_col = "effect_allele",

other_allele_col = "other_allele",

pval_col = "pval",

eaf_col = "eaf")

# outcome modified

gut_dental_out_dat.genus$outcome <- 'GINGIVITIS & PERIODONTAL'

view(gut_dental_out_dat.genus) # obs: 758 comments Duplicated SNPs present

write.csv(gut_dental_out_dat.genus,"gut_dental_out_dat[genus].csv")

# ==================================

##2.4 Outcomes(2) gums bleeding

# ==================================

#3. Outcomes DATA

gut_dental_out_dat.genus2 <- extract_outcome_data(snps = dental_exp_dat_2$SNP, # dental_exp_dat_2$SNP -- microbiota genus

outcomes = 'ukb-b-7872', # 'ukb-b-7872' == Mouth/teeth dental problems: Bleeding gums

maf_threshold = 0.01, #proxies = F,

access_token = NULL)

head(gut_dental_out_dat.genus2) # 789 obs , same as dental_exp_dat_2

write.csv(gut_dental_out_dat.genus2,'gut_dental_out_genus2.csv')

# outcome modified

#view(gut_dental_out_dat.genus2) # obs: 789

#write.csv(gut_dental_out_dat.genus2,"gut_dental_out_dat[genus2].csv")

# Other key points:

# - Sample overlapping

# - Proxy search: https://snipa.helmholtz-muenchen.de/snipa3/

# - Numb. of outcome SNP ?

# - Harmonization: 1. Exposure-SNP vs. Outcome-SNP; 2. EAF palindromic SNP; 3. Exclude incompatible SNP

# ========================================================

## MR/Forest plot step: Outcomes(1) GINGIVITIS_PERIODONTAL

# ========================================================

dental_dat_anal.genus <- harmonise_data(dental_exp_dat_2, gut_dental_out_dat.genus)

write.csv(dental_dat_anal.genus,'harmonise_gut_dental_02[genus].csv')

# add outcome_se

dental_dat_anal.genus$se.outcome=sqrt(((dental_dat_anal.genus$beta.outcome)^2)/qchisq(dental_dat_anal.genus$pval.outcome,1,lower.tail=F))

head(dental_dat_anal.genus)

# revised id

write_xlsx(dental_dat_anal.genus,"02_gut_to_dental_finn[genus].xlsx")

dental_dat_anal.genus %>% dplyr::select(pval.outcome) %>% summary() ## SNP to outcome check

# all results

res_all_dental_anal.genus <- mr(dental_dat_anal.genus, method_list = c("mr_ivw","mr_egger_regression","mr_weighted_median"))

#simple ivw results

res_ivw_dental_anal.genus <- mr(dental_dat_anal.genus, method_list = c("mr_ivw"))

# odds ratios

res_ivw_or_dental_anal.genus <- generate_odds_ratios(res_ivw_dental_anal.genus)

res_all_or_dental_anal.genus <- generate_odds_ratios(res_all_dental_anal.genus)

write.csv(res_ivw_or_dental_anal.genus,'res_ivw_or_dental_anal[genus].csv')

write.csv(res_all_or_dental_anal.genus,'res_all_or_dental_anal[genus].csv')

# Exclude the exposure ID labels from the exposure column

res_ivw_or_dental_anal.genus <- split_outcome(res_ivw_or_dental_anal.genus)

# this sorts results by decreasing effect size (largest effect at top of the plot)http://127.0.0.1:41245/graphics/plot_zoom_png?width=1184&height=969

# Forest plot

res_plot1_dental_anal.genus <- res_ivw_or_dental_anal.genus

plot1_dental_anal.genus <- forest_plot_1_to_many(

res_plot1_dental_anal.genus,

b = "or",

se = "se",

exponentiate = TRUE,

trans = "log2",

ao_slc = FALSE,

lo = 0.6, # beta upci

up = 2, # beta loci

col1_width = 2,

TraitM = "exposure", # exposure/outcome

col_text_size = 3,

shape_points = 15,

xlab = ""

)

plot1_dental_anal.genus

write.csv(res_ivw_or_dental_anal.genus,"res_ivw_or_dental_anal[genus].csv")

## ******************************************

## ------------- statistics -----------------

## -------------- [Genus] ------------------

## ------------- outcomes(1) ----------------

## dental_dat_anal.genus <- harmonise_data()

## ******************************************

# heterogeneity statistics

hetero_dental_dat_anal.genus <- mr_heterogeneity(dental_dat_anal.genus)

mr_heterogeneity(dental_dat_anal.genus, method_list=c("mr_egger_regression", "mr_ivw"))

write.csv(hetero_dental_dat_anal.genus,'hetero_dental_dat_anal.genus.csv')

# pleiotropy

pleio_dental_dat_anal.genus <- mr_pleiotropy_test(dental_dat_anal.genus)

pleio_dental_dat_anal.genus

write.csv(pleio_dental_dat_anal.genus,'pleio_dental_dat_anal.genus.csv')

#leave-one-out

single_dental_dat_anal.genus <- mr_singlesnp(dental_dat_anal.genus)

loo_dental_dat_anal.genus <- mr_leaveoneout(dental_dat_anal.genus)

write.csv(single_dental_dat_anal.genus,'single_dental_dat_anal.genus.csv')

write.csv(loo_dental_dat_anal.genus,'loo_dental_dat_anal.genus.csv')

# heterogeneity statistics

# mr_presso((BetaOutcome = "Y_effect", BetaExposure = "E1_effect",

#SdOutcome = "Y_se", SdExposure = "E1_se", OUTLIERtest = TRUE,

#DISTORTIONtest = TRUE, data = SummaryStats, NbDistribution = 1000, SignifThreshold = 0.05)

# data cleaning

head(res_all_or_dental_anal.genus)

# Scatter plot

# mr_ivw, mr_egger_regression, mr_weighted_median

res_subgroup_dental_dat_anal.genus <- mr(dental_dat_anal.genus,method_list = c("mr_ivw","mr_egger_regression","mr_weighted_median"))

p1_dental_dat_anal.genus <- mr_scatter_plot(res_subgroup_dental_dat_anal.genus, dental_dat_anal.genus)

# plot frame [1]

# p1[[1]]

# 5 IN 1

length(p1_dental_dat_anal.genus)

#p1_dental_dat_anal.family

# export pdf plot

for (i in 1:length(p1_dental_dat_anal.genus)) {

# create PDF

pdf(paste0("plot_genus_", i, ".pdf"))

# Print plots

print(p1_dental_dat_anal.genus[[i]])

# turn off the sequence

dev.off()

}

# Forest plot

res_single_dental_dat_anal.genus <- mr_singlesnp(dental_dat_anal.genus)

head(res_single_dental_dat_anal.genus)

p2_forest_dental_dat_anal.genus <- mr_forest_plot(res_single_dental_dat_anal.genus)

length(p2_forest_dental_dat_anal.genus)

# p2_forest_dental_dat_anal.family

# export pdf plot

for (i in 1:length(p2_forest_dental_dat_anal.genus)) {

# create PDF

pdf(paste0("Forest_plot_", i, ".pdf"))

# Print plots

print(p2_forest_dental_dat_anal.genus[[i]])

# turn off the sequence

dev.off()

}

# Leave-one-out plot - sensitivity

res_loo_dental_dat_anal.genus <- mr_leaveoneout(dental_dat_anal.genus)

p3_loo_dental_dat_anal.genus <- mr_leaveoneout_plot(res_loo_dental_dat_anal.genus)

# p3_loo_dental_dat_anal.genus

# export pdf plot

for (i in 1:length(p3_loo_dental_dat_anal.genus)) {

# create PDF

pdf(paste0("loo_plot_", i, ".pdf"))

# Print plots

print(p3_loo_dental_dat_anal.genus[[i]])

# turn off the sequence

dev.off()

}

# funnel plot

# res_single_dental_dat_anal.family <- mr_singlesnp(dental_dat_anal.family)

res_single_dental_dat_anal.genus <- mr_singlesnp(dental_dat_anal.genus)

p4_funnel_dental_dat_anal.genus <- mr_funnel_plot(res_single_dental_dat_anal.genus)

# p4_funnel_dental_dat_anal.family

# export pdf plot

for (i in 1:length(p4_funnel_dental_dat_anal.genus)) {

# create PDF

pdf(paste0("funnel_", i, ".pdf"))

# Print plots

print(p4_funnel_dental_dat_anal.genus[[i]])

# turn off the sequence

dev.off()

}

## ******************************************

## ******************************************

# ================================================

## MR/Forest plot step: Outcomes(2) Gums bleeding

# ================================================

dental_dat_anal.genus2 <- harmonise_data(dental_exp_dat_2, gut_dental_out_dat.genus2)

head(dental_dat_anal.genus2)

write.csv(dental_dat_anal.genus2,'harmonise_gut_dental_02[genus2].csv')

# add outcome_se

dental_dat_anal.genus2$se.outcome=sqrt(((dental_dat_anal.genus2$beta.outcome)^2)/qchisq(dental_dat_anal.genus2$pval.outcome,1,lower.tail=F))

# revised id

write_xlsx(dental_dat_anal.genus2,"02_gut_to_dental_finn[genus2].xlsx")

dental_dat_anal.genus2 %>% dplyr::select(pval.outcome) %>% summary() ## SNP to outcome check

# all results

res_all_dental_anal.genus2 <- mr(dental_dat_anal.genus2, method_list = c("mr_ivw","mr_egger_regression","mr_weighted_median"))

#simple ivw results

res_ivw_dental_anal.genus2 <- mr(dental_dat_anal.genus2, method_list = c("mr_ivw"))

# odds ratios

res_ivw_or_dental_anal.genus2 <- generate_odds_ratios(res_ivw_dental_anal.genus2)

res_all_or_dental_anal.genus2 <- generate_odds_ratios(res_all_dental_anal.genus2)

write.csv(res_ivw_or_dental_anal.genus2,'res_ivw_or_dental_anal[genus2].csv')

write.csv(res_all_or_dental_anal.genus2,'res_all_or_dental_anal[genus2].csv')

# Exclude the exposure ID labels from the exposure column

res_ivw_or_dental_anal.genus2 <- split_outcome(res_ivw_or_dental_anal.genus2)

# this sorts results by decreasing effect size (largest effect at top of the plot)http://127.0.0.1:41245/graphics/plot_zoom_png?width=1184&height=969

# Forest plot

res_plot1_dental_anal.genus2 <- res_ivw_or_dental_anal.genus2

plot1_dental_anal.genus2 <- forest_plot_1_to_many(

res_plot1_dental_anal.genus2,

b = "or",

se = "se",

exponentiate = TRUE,

trans = "log2",

ao_slc = FALSE,

lo = 0.95, # beta upci

up = 1.05, # beta loci

col1_width = 2,

TraitM = "exposure", # exposure/outcome

col_text_size = 3,

shape_points = 15,

xlab = ""

)

plot1_dental_anal.genus2

write.csv(res_ivw_dental_anal.genus2, "res_ivw_dental_anal[genus2].csv")

## ******************************************

## ------------- statistics -----------------

## -------------- [Genus2] ------------------

## ------------- outcomes(2) ----------------

## dental_dat_anal.genus2 <- harmonise_data()

## ******************************************

# heterogeneity statistics

hetero_dental_dat_anal.genus2 <- mr_heterogeneity(dental_dat_anal.genus2)

mr_heterogeneity(dental_dat_anal.genus2, method_list=c("mr_egger_regression", "mr_ivw"))

write.csv(hetero_dental_dat_anal.genus2,'hetero_dental_dat_anal.genus2.csv')

# pleiotropy

pleio_dental_dat_anal.genus2 <- mr_pleiotropy_test(dental_dat_anal.genus2)

pleio_dental_dat_anal.genus2

write.csv(pleio_dental_dat_anal.genus2,'pleio_dental_dat_anal.genus2.csv')

#leave-one-out

single_dental_dat_anal.genus2 <- mr_singlesnp(dental_dat_anal.genus2)

loo_dental_dat_anal.genus2 <- mr_leaveoneout(dental_dat_anal.genus2)

write.csv(single_dental_dat_anal.genus2,'single_dental_dat_anal.genus2.csv')

write.csv(loo_dental_dat_anal.genus2,'loo_dental_dat_anal.genus2.csv')

# heterogeneity statistics

# mr_presso((BetaOutcome = "Y_effect", BetaExposure = "E1_effect",

#SdOutcome = "Y_se", SdExposure = "E1_se", OUTLIERtest = TRUE,

#DISTORTIONtest = TRUE, data = SummaryStats, NbDistribution = 1000, SignifThreshold = 0.05)

# data cleaning

head(res_all_or_dental_anal.genus2)

# Scatter plot

# mr_ivw, mr_egger_regression, mr_weighted_median

res_subgroup_dental_dat_anal.genus2 <- mr(dental_dat_anal.genus2,method_list = c("mr_ivw","mr_egger_regression","mr_weighted_median"))

res_subgroup_dental_dat_anal.genus2$outcome <- 'Bleeding gums'

#res_subgroup_dental_dat_anal.genus2 <- res_subgroup_dental_dat_anal.genus2 %>% dplyr::filter(nsnp > 4)

head(res_subgroup_dental_dat_anal.genus2) #dental_exp_dat_2

head(dental_dat_anal.genus2)

# exclude 'ebi-a-GCST90017023'

res_dental_dat_filter.genus2 <- res_subgroup_dental_dat_anal.genus2 %>% dplyr::filter(id.exposure != 'ebi-a-GCST90017023')

dental_dat_filter.genus2 <- dental_dat_anal.genus2 %>% dplyr::filter(id.exposure != 'ebi-a-GCST90017023')

p1_dental_dat_anal.genus2 <- mr_scatter_plot(res_dental_dat_filter.genus2, dental_dat_filter.genus2)

# plot frame [1]

# p1[[1]]

# 5 IN 1

length(p1_dental_dat_anal.genus2)

#p1_dental_dat_anal.family

# export pdf plot

for (i in 1:length(p1_dental_dat_anal.genus2)) {

# create PDF

pdf(paste0("plot_genus2_", i, ".pdf"))

# Print plots

print(p1_dental_dat_anal.genus2[[i]])

# turn off the sequence

dev.off()

}

# Forest plot

res_single_dental_dat_anal.genus2 <- mr_singlesnp(dental_dat_anal.genus2)

head(res_single_dental_dat_anal.genus2)

p2_forest_dental_dat_anal.genus2 <- mr_forest_plot(res_single_dental_dat_anal.genus2)

length(p2_forest_dental_dat_anal.genus2)

# p2_forest_dental_dat_anal.family

# export pdf plot

for (i in 1:length(p2_forest_dental_dat_anal.genus2)) {

# create PDF

pdf(paste0("Forest_plot_", i, ".pdf"))

# Print plots

print(p2_forest_dental_dat_anal.genus2[[i]])

# turn off the sequence

dev.off()

}

# Leave-one-out plot - sensitivity

res_loo_dental_dat_anal.genus2 <- mr_leaveoneout(dental_dat_anal.genus2)

p3_loo_dental_dat_anal.genus2 <- mr_leaveoneout_plot(res_loo_dental_dat_anal.genus2)

# p3_loo_dental_dat_anal.genus2

# export pdf plot

for (i in 1:length(p3_loo_dental_dat_anal.genus2)) {

# create PDF

pdf(paste0("loo_plot_", i, ".pdf"))

# Print plots

print(p3_loo_dental_dat_anal.genus2[[i]])

# turn off the sequence

dev.off()

}

# funnel plot

# res_single_dental_dat_anal.family <- mr_singlesnp(dental_dat_anal.family)

res_single_dental_dat_anal.genus2 <- mr_singlesnp(dental_dat_anal.genus2)

p4_funnel_dental_dat_anal.genus2 <- mr_funnel_plot(res_single_dental_dat_anal.genus2)

# p4_funnel_dental_dat_anal.family

# export pdf plot

for (i in 1:length(p4_funnel_dental_dat_anal.genus2)) {

# create PDF

pdf(paste0("funnel_", i, ".pdf"))

# Print plots

print(p4_funnel_dental_dat_anal.genus2[[i]])

# turn off the sequence

dev.off()

}

## ******************************************

# A bidirectional MR

## ******************************************

#*********************************************************************#

# online - exposure extraction

# ukb-b-7872 == Bleeding gums

# RESULTS == NULL!!!!!!!!!!!!!!!!!!!!!!!!!!!!!!!!!!!!!!!!!!!!!!!!!

#*********************************************************************#

#************************

#pval.exposure < 5e-6,ebi-a-

#************************

dental_rev_ukb_exp2 <- extract_instruments(outcomes = c("ukb-b-7872"), # 'ukb-b-7872' == Bleeding gums

p1 = 5e-06,

clump = TRUE,

r2 = 0.001,

kb = 10000,

access_token = ieugwasr::check_access_token(),

force_server = FALSE )

#=================================

# gut microbiota [family]

#=================================

dental_gut_out.family2 <- extract_outcome_data(snps = dental_rev_ukb_exp2$SNP, # 'ukb-b-7872' == Bleeding gums

outcomes = 'ebi-a-GCST90016936', # microbiota

maf_threshold = 0.01, # proxies = F,

access_token = NULL)

head(dental_gut_out.family2)

write_xlsx(dental_gut_out.family2,"dental_gut_out.family2.xlsx") # exposure: dental_rev_ukb_exp2

family_Gumbleeding_to_microbiota <- harmonise_data(dental_rev_ukb_exp2, dental_gut_out.family2) #Gumbleeding_to_microbiota

family_Gumbleeding_to_microbiota$se.outcome=sqrt(((family_Gumbleeding_to_microbiota$beta.outcome)^2)/

qchisq(family_Gumbleeding_to_microbiota$pval.outcome,1,lower.tail=F))

write_xlsx(family_Gumbleeding_to_microbiota,"family_Gumbleeding_to_microbiota.xlsx")

res_all_family_GB_M <- mr(family_Gumbleeding_to_microbiota,method_list = c("mr_ivw","mr_egger_regression","mr_weighted_median"))

res_all_family_GB_M <- generate_odds_ratios(res_all_family_GB_M)

#simple ivw results

res_ivw_family_GB_M <- mr(family_Gumbleeding_to_microbiota,method_list = c("mr_ivw"))

res_ivw_family_GB_M <- generate_odds_ratios(res_ivw_family_GB_M)

write_xlsx(res_all_family_GB_M,"res_all_family_GB_M.xlsx")

write_xlsx(res_ivw_family_GB_M,"res_ivw_family_GB_M.xlsx")

# subset filter: up-down

subset_on_method(res_ivw_family_GB_M)

# heterogeneity statistics

hetero_family_Gumbleeding_to_microbiota <- mr_heterogeneity(family_Gumbleeding_to_microbiota)

hetero_family_Gumbleeding_to_microbiota_ivoregg <- mr_heterogeneity(family_Gumbleeding_to_microbiota,

method_list=c("mr_egger_regression", "mr_ivw"))

write_xlsx(hetero_family_Gumbleeding_to_microbiota,"hetero_family_Gumbleeding_to_microbiota.xlsx")

# pleiotropy

pleio_family_GBtoMic <-mr_pleiotropy_test(family_Gumbleeding_to_microbiota)

pleio_family_GBtoMic

write_xlsx(pleio_family_GBtoMic,"pleio_family_GBtoMic.xlsx")

#leave-one-out

res_single_family_GBtoMic <- mr_singlesnp(family_Gumbleeding_to_microbiota)

res_single_family_GBtoMic

res_loo_family_GBtoMic <- mr_leaveoneout(family_Gumbleeding_to_microbiota)

res_loo_family_GBtoMic

# Scatter plot

# mr_ivw, mr_egger_regression, mr_weighted_median

p1_family_GB_M <- mr_scatter_plot(res_all_family_GB_M, family_Gumbleeding_to_microbiota)

# plot frame [1]

# p1[[1]]

# 5 IN 1

length(p1_family_GB_M)

# export pdf plot

for (i in 1:length(p1_family_GB_M)) {

# create PDF

pdf(paste0("reg_family_GB_M", i, ".pdf"))

# Print plots

print(p1_family_GB_M[[i]])

# turn off the sequence

dev.off()

}

# Forest plot

res_single_family_GB_M <- mr_singlesnp(family_Gumbleeding_to_microbiota)

p2_family_GB_M <- mr_forest_plot(res_single_family_GB_M)

# export pdf plot

for (i in 1:length(p2_family_GB_M)) {

# create PDF

pdf(paste0("Forest_family_GB_M", i, ".pdf"))

# Print plots

print(p2_family_GB_M[[i]])

# turn off the sequence

dev.off()

}

# Leave-one-out plot - sensitivity

res_loo_family_GB_M <- mr_leaveoneout(family_Gumbleeding_to_microbiota)

p3_family_GB_M <- mr_leaveoneout_plot(res_loo_family_GB_M)

# export pdf plot

for (i in 1:length(p3_family_GB_M)) {

# create PDF

pdf(paste0("LOO_family_GB_M", i, ".pdf"))

# Print plots

print(p3_family_GB_M[[i]])

# turn off the sequence

dev.off()

}

# funnel plot

res_single_family_GB_M <- mr_singlesnp(family_Gumbleeding_to_microbiota)

p4_family_GB_M <- mr_funnel_plot(res_single_family_GB_M)

# export pdf plot

for (i in 1:length(p4_family_GB_M)) {

# create PDF

pdf(paste0("funnel_family_GB_M", i, ".pdf"))

# Print plots

print(p4_family_GB_M[[i]])

# turn off the sequence

dev.off()

}

#=================================

# gut microbiota [genus]

#=================================

dental_gut_out.genus2 <- extract_outcome_data(snps = dental_rev_ukb_exp2$SNP, # 'ukb-b-7872' == Bleeding gums

outcomes = c('ebi-a-GCST90017006', # microbiota

'ebi-a-GCST90017009',

'ebi-a-GCST90017016',

'ebi-a-GCST90017032',

'ebi-a-GCST90017043',

'ebi-a-GCST90017044',

'ebi-a-GCST90017047'),

maf_threshold = 0.01, # proxies = F,

access_token = NULL)

head(dental_gut_out.genus2)

write_xlsx(dental_gut_out.genus2,"dental_gut_out.genus2.xlsx") # exposure: dental_rev_ukb_exp2

genus_Gumbleeding_to_microbiota <- harmonise_data(dental_rev_ukb_exp2, dental_gut_out.genus2) #Gumbleeding_to_microbiota

genus_Gumbleeding_to_microbiota$se.outcome=sqrt(((genus_Gumbleeding_to_microbiota$beta.outcome)^2)/

qchisq(genus_Gumbleeding_to_microbiota$pval.outcome,1,lower.tail=F))

write_xlsx(genus_Gumbleeding_to_microbiota,"genus_Gumbleeding_to_microbiota.xlsx")

res_all_genus_GB_M <- mr(genus_Gumbleeding_to_microbiota,method_list = c("mr_ivw","mr_egger_regression","mr_weighted_median"))

res_all_genus_GB_M <- generate_odds_ratios(res_all_genus_GB_M)

#simple ivw results

res_ivw_genus_GB_M <- mr(genus_Gumbleeding_to_microbiota,method_list = c("mr_ivw"))

res_ivw_genus_GB_M <- generate_odds_ratios(res_ivw_GB_M)

write_xlsx(res_all_genus_GB_M,"res_all_genus_GB_M.xlsx")

write_xlsx(res_ivw_genus_GB_M,"res_ivw_genus_GB_M.xlsx")

# subset filter: up-down

subset_on_method(res_ivw_genus_GB_M)

# heterogeneity statistics

hetero_genus_Gumbleeding_to_microbiota <- mr_heterogeneity(genus_Gumbleeding_to_microbiota)

hetero_genus_Gumbleeding_to_microbiota_ivoregg <- mr_heterogeneity(genus_Gumbleeding_to_microbiota,

method_list=c("mr_egger_regression", "mr_ivw"))

write_xlsx(hetero_genus_Gumbleeding_to_microbiota,"hetero_genus_Gumbleeding_to_microbiota.xlsx")

# pleiotropy

pleio_genus_GBtoMic <-mr_pleiotropy_test(genus_Gumbleeding_to_microbiota)

pleio_genus_GBtoMic

write_xlsx(pleio_genus_GBtoMic,"pleio_genus_GBtoMic.xlsx")

#leave-one-out

res_single_genus_GBtoMic <- mr_singlesnp(genus_Gumbleeding_to_microbiota)

res_single_genus_GBtoMic

res_loo_genus_GBtoMic <- mr_leaveoneout(genus_Gumbleeding_to_microbiota)

res_loo_genus_GBtoMic

# Scatter plot

# mr_ivw, mr_egger_regression, mr_weighted_median

p1_genus_GB_M <- mr_scatter_plot(res_all_genus_GB_M, genus_Gumbleeding_to_microbiota)

# plot frame [1]

# p1[[1]]

# 5 IN 1

length(p1_genus_GB_M)

# export pdf plot

for (i in 1:length(p1_genus_GB_M)) {

# create PDF

pdf(paste0("reg_genus_GB_M", i, ".pdf"))

# Print plots

print(p1_genus_GB_M[[i]])

# turn off the sequence

dev.off()

}

# Forest plot

res_single_genus_GB_M <- mr_singlesnp(genus_Gumbleeding_to_microbiota)

p2_genus_GB_M <- mr_forest_plot(res_single_genus_GB_M)

# export pdf plot

for (i in 1:length(p2_genus_GB_M)) {

# create PDF

pdf(paste0("Forest_genus_GB_M", i, ".pdf"))

# Print plots

print(p2_genus_GB_M[[i]])

# turn off the sequence

dev.off()

}

# Leave-one-out plot - sensitivity

res_loo_genus_GB_M <- mr_leaveoneout(genus_Gumbleeding_to_microbiota)

p3_genus_GB_M <- mr_leaveoneout_plot(res_loo_genus_GB_M)

# export pdf plot

for (i in 1:length(p3_genus_GB_M)) {

# create PDF

pdf(paste0("LOO_genus_GB_M", i, ".pdf"))

# Print plots

print(p3_genus_GB_M[[i]])

# turn off the sequence

dev.off()

}

# funnel plot

res_single_genus_GB_M <- mr_singlesnp(genus_Gumbleeding_to_microbiota)

p4_genus_GB_M <- mr_funnel_plot(res_single_genus_GB_M)

# export pdf plot

for (i in 1:length(p4_genus_GB_M)) {

# create PDF

pdf(paste0("funnel_genus_GB_M", i, ".pdf"))

# Print plots

print(p4_genus_GB_M[[i]])

# turn off the sequence

dev.off()

}

#*********************************************************************#

# Offline - exposure extraction

# finngen_R9_K11_GINGIVITIS_PERIODONTAL

# RESULTS == NULL!!!!!!!!!!!!!!!!!!!!!!!!!!!!!!!!!!!!!!!!!!!!!!!!!

#*********************************************************************#

# ----------------------------------------------------------------------------

# Bidirectional MR study

# Reverse exosure (1) -- finngen_R9_K11_GINGIVITIS_PERIODONTAL -- offline

# LINE 150

# ----------------------------------------------------------------------------

dental_GWAS_1 <- read.delim("finngen_R9_K11_GINGIVITIS_PERIODONTAL.gz") # txt | txt.gz | tsv.gz

head(dental_GWAS_1) # finngen_R9_K11_GINGIVITIS_PERIODONTAL

dental_GWAS_1.filter <- dplyr::filter(dental_GWAS_1, pval < 1e-5) # filtered data: dental_GWAS_1

nrow(dental_GWAS_1.filter)

View(dental_GWAS_1.filter)

write.csv(dental_GWAS_1.filter,"dental_GWAS_1.filter.csv")

head(dental_GWAS_1.filter)

#delete first col!

dental_rev_finn_exp1 <- read_exposure_data(filename = "dental_GWAS_1.filter.csv",

clump = FALSE,

sep = ",",

phenotype_col = "Phenotype",

snp_col = "rsids",

beta_col = "beta",

se_col = "sebeta",

eaf_col = "af_alt",

effect_allele_col = "alt",

other_allele_col = "ref",

pval_col = "pval",

chr_col = "X.chrom",

pos_col = "pos")

dental_rev_finn_exp1$exposure <- "GINGIVITIS_PERIODONTAL"

head(dental_rev_finn_exp1)

write.csv(dental_rev_finn_exp1,"dental_rev_finn_exp1.csv")

dental_rev_finn_exp1_clumped <- clump_data(dental_rev_finn_exp1,

clump_kb = 10000,

clump_r2 = 0.001,

clump_p1 = 1,

clump_p2 = 1,

pop = "EUR")

head(dental_rev_finn_exp1_clumped)

#************************

#pval.exposure < 5e-6

#************************

dental_rev_finn_exp1_clumped.pval <- dplyr::filter(dental_rev_finn_exp1_clumped,pval.exposure < 5e-6)

#dental_rev_finn_exp1_clumped.pval$exposure <- "GINGIVITIS_PERIODONTAL"

head(dental_rev_finn_exp1_clumped.pval)

#=================================

# gut microbiota [family]

# exosure: dental_rev_finn_exp1_clumped.pval

#=================================

dental_gut_out.family1 <- extract_outcome_data(snps = dental_rev_finn_exp1_clumped.pval$SNP, # ukb-b-7872 == Bleeding gums

outcomes = 'ebi-a-GCST90016936', # microbiota

maf_threshold = 0.01, # proxies = F,

access_token = NULL)

head(dental_gut_out.family1)

write_xlsx(dental_gut_out.family1,"dental_gut_out.family1.xlsx") # exposure: dental_rev_finn_exp1_clumped.pval

family_PERIODONTAL_to_microbiota <- harmonise_data(dental_rev_finn_exp1_clumped.pval, dental_gut_out.family1) #PERIODONTAL_to_microbiota

family_PERIODONTAL_to_microbiota$se.outcome=sqrt(((family_PERIODONTAL_to_microbiota$beta.outcome)^2)/

qchisq(family_PERIODONTAL_to_microbiota$pval.outcome,1,lower.tail=F))

head(family_PERIODONTAL_to_microbiota)

write_xlsx(family_PERIODONTAL_to_microbiota,"family_PERIODONTAL_to_microbiota.xlsx")

res_all_family_PERIO_M <- mr(family_PERIODONTAL_to_microbiota,method_list = c("mr_ivw","mr_egger_regression","mr_weighted_median"))

res_all_family_PERIO_M <- generate_odds_ratios(res_all_family_PERIO_M)

#simple ivw results

res_ivw_family_PERIO_M <- mr(family_PERIODONTAL_to_microbiota,method_list = c("mr_ivw"))

res_ivw_family_PERIO_M <- generate_odds_ratios(res_ivw_family_PERIO_M)

write_xlsx(res_all_family_PERIO_M,"res_all_family_PERIO_M.xlsx")

write_xlsx(res_ivw_family_PERIO_M,"res_ivw_family_PERIO_M.xlsx")

# subset filter: up-down

subset_on_method(res_ivw_family_PERIO_M)

# heterogeneity statistics

hetero_family_PERIODONTAL_to_microbiota <- mr_heterogeneity(family_PERIODONTAL_to_microbiota)

hetero_family_PERIODONTAL_to_microbiota_ivoregg <- mr_heterogeneity(family_PERIODONTAL_to_microbiota,

method_list=c("mr_egger_regression", "mr_ivw"))

write_xlsx(hetero_family_PERIODONTAL_to_microbiota,"hetero_family_PERIODONTAL_to_microbiota.xlsx")

# pleiotropy

pleio_family_PERIOtoMic <-mr_pleiotropy_test(family_PERIODONTAL_to_microbiota)

pleio_family_PERIOtoMic

write_xlsx(pleio_family_PERIOtoMic,"pleio_family_PERIOtoMic.xlsx")

#leave-one-out

res_single_family_PERIOtoMic <- mr_singlesnp(family_PERIODONTAL_to_microbiota)

res_single_family_PERIOtoMic

res_loo_family_PERIOtoMic <- mr_leaveoneout(family_PERIODONTAL_to_microbiota)

res_loo_family_PERIOtoMic

# Scatter plot

# mr_ivw, mr_egger_regression, mr_weighted_median

p1_family_PERIO_M <- mr_scatter_plot(res_all_family_PERIO_M, family_PERIODONTAL_to_microbiota)

# plot frame [1]

# p1[[1]]

# 5 IN 1

length(p1_family_PERIO_M)

# export pdf plot

for (i in 1:length(p1_family_PERIO_M)) {

# create PDF

pdf(paste0("reg_family_PERIO_M", i, ".pdf"))

# Print plots

print(p1_family_PERIO_M[[i]])

# turn off the sequence

dev.off()

}

# Forest plot

res_single_family_PERIO_M <- mr_singlesnp(family_PERIODONTAL_to_microbiota)

p2_family_PERIO_M <- mr_forest_plot(res_single_family_PERIO_M)

# export pdf plot

for (i in 1:length(p2_family_PERIO_M)) {

# create PDF

pdf(paste0("Forest_family_PERIO_M", i, ".pdf"))

# Print plots

print(p2_family_PERIO_M[[i]])

# turn off the sequence

dev.off()

}

# Leave-one-out plot - sensitivity

res_loo_family_PERIO_M <- mr_leaveoneout(family_PERIODONTAL_to_microbiota)

p3_family_PERIO_M <- mr_leaveoneout_plot(res_loo_family_PERIO_M)

# export pdf plot

for (i in 1:length(p3_family_PERIO_M)) {

# create PDF

pdf(paste0("LOO_family_PERIO_M", i, ".pdf"))

# Print plots

print(p3_family_PERIO_M[[i]])

# turn off the sequence

dev.off()

}

# funnel plot

res_single_family_PERIO_M <- mr_singlesnp(family_PERIODONTAL_to_microbiota)

p4_family_PERIO_M <- mr_funnel_plot(res_single_family_PERIO_M)

# export pdf plot

for (i in 1:length(p4_family_PERIO_M)) {

# create PDF

pdf(paste0("funnel_family_PERIO_M", i, ".pdf"))

# Print plots

print(p4_family_PERIO_M[[i]])

# turn off the sequence

dev.off()

}

#=================================

# gut microbiota [genus]

#=================================

dental_gut_out.genus1 <- extract_outcome_data(snps = dental_rev_finn_exp1_clumped.pval$SNP, # 'ukb-b-7872' == Bleeding gums

outcomes = c('ebi-a-GCST90017006', # microbiota

'ebi-a-GCST90017009',

'ebi-a-GCST90017016',

'ebi-a-GCST90017032',

'ebi-a-GCST90017043',

'ebi-a-GCST90017044',

'ebi-a-GCST90017047'),

maf_threshold = 0.01, # proxies = F,

access_token = NULL)

head(dental_gut_out.genus1)

write_xlsx(dental_gut_out.genus1,"dental_gut_out.genus1.xlsx") # exposure: dental_rev_finn_exp1_clumped.pval

genus_PERIODONTAL_to_microbiota <- harmonise_data(dental_rev_finn_exp1_clumped.pval, dental_gut_out.genus1) #PERIODONTAL_to_microbiota

genus_PERIODONTAL_to_microbiota$se.outcome=sqrt(((genus_PERIODONTAL_to_microbiota$beta.outcome)^2)/

qchisq(genus_PERIODONTAL_to_microbiota$pval.outcome,1,lower.tail=F))

head(genus_PERIODONTAL_to_microbiota)

write_xlsx(genus_PERIODONTAL_to_microbiota,"genus_PERIODONTAL_to_microbiota.xlsx")

res_all_genus_PERIO_M <- mr(genus_PERIODONTAL_to_microbiota,method_list = c("mr_ivw","mr_egger_regression","mr_weighted_median"))

res_all_genus_PERIO_M <- generate_odds_ratios(res_all_genus_PERIO_M)

#simple ivw results

res_ivw_genus_PERIO_M <- mr(genus_PERIODONTAL_to_microbiota,method_list = c("mr_ivw"))

res_ivw_genus_PERIO_M <- generate_odds_ratios(res_ivw_genus_PERIO_M)

write_xlsx(res_all_genus_PERIO_M,"res_all_genus_PERIO_M.xlsx")

write_xlsx(res_ivw_genus_PERIO_M,"res_ivw_genus_PERIO_M.xlsx")

# subset filter: up-down

subset_on_method(res_ivw_genus_PERIO_M)

# heterogeneity statistics

hetero_genus_PERIODONTAL_to_microbiota <- mr_heterogeneity(genus_PERIODONTAL_to_microbiota)

hetero_genus_PERIODONTAL_to_microbiota_ivoregg <- mr_heterogeneity(genus_PERIODONTAL_to_microbiota,

method_list=c("mr_egger_regression", "mr_ivw"))

write_xlsx(hetero_genus_PERIODONTAL_to_microbiota,"hetero_genus_PERIODONTAL_to_microbiota.xlsx")

# pleiotropy

pleio_genus_PERIOtoMicro <-mr_pleiotropy_test(genus_PERIODONTAL_to_microbiota)

pleio_genus_PERIOtoMicro

write_xlsx(pleio_genus_PERIOtoMicro,"pleio_genus_PERIOtoMicro.xlsx")

#leave-one-out

res_single_genus_PERIOtoMicro <- mr_singlesnp(genus_PERIODONTAL_to_microbiota)

res_single_genus_PERIOtoMicro

res_loo_genus_PERIOtoMicro <- mr_leaveoneout(genus_PERIODONTAL_to_microbiota)

res_loo_genus_PERIOtoMic

# Scatter plot

# mr_ivw, mr_egger_regression, mr_weighted_median

p1_genus_PERIO_M <- mr_scatter_plot(res_all_genus_PERIO_M, genus_PERIODONTAL_to_microbiota)

# plot frame [1]

# p1[[1]]

# 5 IN 1

length(p1_genus_PERIO_M)

# export pdf plot

for (i in 1:length(p1_genus_PERIO_M)) {

# create PDF

pdf(paste0("reg_genus_PERIO_M", i, ".pdf"))

# Print plots

print(p1_genus_PERIO_M[[i]])

# turn off the sequence

dev.off()

}

# Forest plot

res_single_genus_PERIO_M <- mr_singlesnp(genus_PERIODONTAL_to_microbiota)

p2_genus_PERIO_M <- mr_forest_plot(res_single_genus_PERIO_M)

# export pdf plot

for (i in 1:length(p2_genus_PERIO_M)) {

# create PDF

pdf(paste0("Forest_genus_PERIO_M", i, ".pdf"))

# Print plots

print(p2_genus_PERIO_M[[i]])

# turn off the sequence

dev.off()

}

# Leave-one-out plot - sensitivity

res_loo_genus_PERIO_M <- mr_leaveoneout(genus_PERIODONTAL_to_microbiota)

p3_genus_PERIO_M <- mr_leaveoneout_plot(res_loo_genus_PERIO_M)

# export pdf plot

for (i in 1:length(p3_genus_PERIO_M)) {

# create PDF

pdf(paste0("LOO_genus_PERIO_M", i, ".pdf"))

# Print plots

print(p3_genus_PERIO_M[[i]])

# turn off the sequence

dev.off()

}

# funnel plot

res_single_genus_PERIO_M <- mr_singlesnp(genus_PERIODONTAL_to_microbiota)

p4_genus_PERIO_M <- mr_funnel_plot(res_single_genus_PERIO_M)

# export pdf plot

for (i in 1:length(p4_genus_PERIO_M)) {

# create PDF

pdf(paste0("funnel_genus_PERIO_M", i, ".pdf"))

# Print plots

print(p4_genus_PERIO_M[[i]])

# turn off the sequence

dev.off()

}
